# Role of casein kinase 1 in the amoeboid migration of B-cell leukemic and lymphoma cells: A quantitative live imaging in the confined environment

**DOI:** 10.3389/fcell.2022.911966

**Published:** 2022-12-06

**Authors:** Štěpán Čada, Olga Vondálová Blanářová, Kristína Gömoryová, Antónia Mikulová, Petra Bačovská, Nikodém Zezula, Alka Kumari Jadaun, Pavlína Janovská, Hana Plešingerová, Vítězslav Bryja

**Affiliations:** ^1^ Department of Experimental Biology, Faculty of Science, Masaryk University, Brno, Czechia; ^2^ Department of Internal Medicine—Hematology and Oncology, University Hospital Brno, Brno, Czechia; ^3^ Department of Cytokinetics, Institute of Biophysics, Academy of Sciences of the Czech Republic, Brno, Czechia

**Keywords:** amoeboid cell migration, chronic lymphocytic leukemia, mantle cell lymphoma, casein kinase 1, live imaging, B cells, uropod

## Abstract

The migratory properties of leukemic cells are commonly associated with their pathological potential and can significantly affect the disease progression. While the research in immunopathology mostly employed powerful indirect methods such as flow cytometry, these cells were rarely observed directly using live imaging microscopy. This is especially true for the malignant cells of the B-cell lineage, such as those originating from chronic lymphocytic leukemia (CLL) and mantle cell lymphoma (MCL). In this study, we employed open-source image analysis tools to automatically and quantitatively describe the amoeboid migration of four B-cell leukemic and lymphoma cell lines and primary CLL cells. To avoid the effect of the shear stress of the medium on these usually non-adherent cells, we have confined the cells using a modified under-agarose assay. Surprisingly, the behavior of tested cell lines differed substantially in terms of basal motility or response to chemokines and VCAM1 stimulation. Since casein kinase 1 (CK1) was reported as a regulator of B-cell migration and a promoter of CLL, we looked at the effects of CK1 inhibition in more detail. Migration analysis revealed that CK1 inhibition induced rapid negative effects on the migratory polarity of these cells, which was quantitatively and morphologically distinct from the effect of ROCK inhibition. We have set up an assay that visualizes endocytic vesicles in the uropod and facilitates morphological analysis. This assay hints that the effect of CK1 inhibition might be connected to defects in polarized intracellular transport. In summary, 1) we introduce and validate a pipeline for the imaging and quantitative assessment of the amoeboid migration of CLL/MCL cells, 2) we provide evidence that the assay is sensitive enough to mechanistically study migration defects identified by the transwell assay, and 3) we describe the polarity defects induced by inhibition or deletion of CK1ε.

## Introduction

Many of the malignancies originating from the B-cell lineage hijack strategies that are required from healthy cells to successfully pass the clonal selection phase during the germinal center (GC) reaction. Apart from aberrant activation of the B-cell receptor (BCR), which simulates successful antigen binding and results in prolonged survival of the blasts, there are other strategies that can increase the chances of precancerous lymphocytes evading immune system regulation (Mlynarczyk et al., 2019). In chronic lymphocytic leukemia (CLL) and mantle cell lymphoma (MCL), for example, the majority of patient samples show aberrant expression of receptor tyrosine kinase-like orphan receptor 1 (ROR1) (Baskar et al., 2008; Barna et al., 2011), a receptor of beta-catenin-independent Wnt signaling (Fukuda et al., 2008). Apart from its importance for the survival of the CLL cells, highlighted by studies employing gene expression silencing and targeting by antibodies (Choudhury et al., 2010; Daneshmanesh et al., 2012), other studies have shown it can affect the cytoskeletal and migratory properties of the leukemic cells (Yu et al., 2015; Hasan et al., 2019).

Migratory properties of leukemic and lymphoma cells of the B-cell lineage have only been so far partially studied despite the fact that efficient migration and chemotaxis are important prerequisites for survival and maturation of healthy B lymphocytes in the GC. The migratory behavior of B cells is at its peak during this process. Immature B cells called centroblasts and centrocytes guided by chemokines circulate between the dark and light zones in the lymph nodes (Mesin et al., 2016). *In vivo* experiments have shown that centroblasts and centrocytes are more polarized and show enhanced migration in comparison with naïve lymphocytes or their mature successors (Allen et al., 2007; Hauser et al., 2007). Interestingly, centroblasts and centrocytes show increased expression of Wnt5a, a known ligand of ROR1 (Janovska et al., 2016). Wnt5a is also produced by the GC microenvironment, represented by follicular dendritic cells (FDCs) (Kim et al., 2012). Strikingly, increased expression of Wnt5a and some other components of beta-catenin-independent signaling is associated with changes in chemotactic and migratory properties and is a predictor of clinical outcome in CLL (Kaucká et al., 2013; Janovska et al., 2016). The aberrant activation of Wnt5a signaling in CLL can be pharmacologically targeted by the inhibitors of casein kinase 1 (CK1) (Janovská et al., 2020). One of the key biological effects of CK1 inhibition is the effective attenuation of the migratory and chemotactic properties of CLL cells (Kaucká et al., 2015; Janovska et al., 2018).

Despite these findings, migration of CLL or MCL cells has so far been described mostly by indirect endpoint methods such as the Boyden chamber (transwell) assay (Boyden, 1962; Jarvis et al., 1976; Burger et al., 1999; Hoellenriegel et al., 2011; Kaucká et al., 2013) or *via* imaging in the non-confined environment (Hutchinson et al., 2014; Kaucká et al., 2015; Malet-Engra et al., 2015; Mele et al., 2018; Dampmann et al., 2020). However, recent studies have highlighted the importance of spatial confinement for the migration of the amoeboid cells (Jacobelli et al., 2010; Liu et al., 2015). Furthermore, given the naturally confining character of the GC microenvironment, in which a high number of developing B cells compete by means of migration, the factor of confinement should not be omitted if we want to construct a credible *in vitro* model. We have thus decided to implement an experimental system that will provide both spatial confinement and, at the same time, limit some of the artifacts often occurring in the direct migration analysis of the low-adhesive cells caused, for example, by the shear stress of the medium. We have optimized a modification of the under-agarose assay and tested its suitability for the analysis of migratory parameters on a panel of B-lymphocyte cell lines originating from CLL and MCL. We have connected this experimental system to the open-source analytical pipeline that allowed us to track and quantify the migratory and phenotypic properties of these cell lines. We believe that our study provides an important reference point for live imaging-based studies of amoeboid migration of normal and transformed B lymphocytes. Furthermore, using this method, we were able to show effects of CK1 inhibition distinct from those of inhibition of another established component of noncanonical Wnt signaling: RhoA–Rho-associated kinase (ROCK). This observation could be a first step toward elucidating the mechanistic role of CK1 in cell migration.

## Results

### Setup of the experimental system for the analysis of the confined migration of chronic lymphocytic leukemia and mantle cell lymphoma cell lines

**FIGURE 1:**
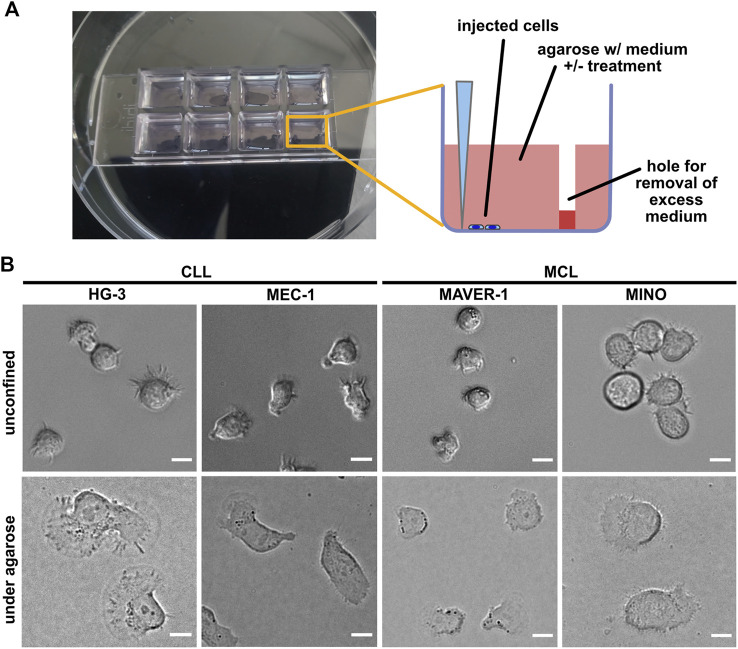
Experimental system for analysis of ameboid migration of leukemic cell lines. **(A)** Scheme of the under-agarose assay experimental system used for migration analysis. The cells are injected under the gel using a 10-μl pipette at the side of the well opposite to hole punched in advance, which serves to collect excess medium. The collected medium needs to be removed before imaging to achieve efficient confinement. Inhibitors or chemokines are added to the gelation mixture before the gel is poured into the wells. **(B)** Representative images of cell lines used in this study. Upper line: cell phenotypes in the normal 2D culture. Lower line: cell phenotypes in the under-agarose assay. Scale bar: 10 μm. Representative videos of cell line behavior under confinement are located in Supplementary Movies S1–S4.

In order to study the migration of B-lymphocyte cell lines in the confined environment, we have been able to set up a modification of the previously reported under-agarose assay protocol (Hons et al., 2018). The experimental setup based on the injection of cells at the bottom of wells filled with agarose is schematized in Figure 1A (see Material and methods for details). Our experimental protocol seems to be highly reproducible (see below), and the key parameters (the efficiency of the injection and the cell spreading) can be easily monitored using a light microscope.

In this study, we have decided to study a panel of four cell lines, two of which are of CLL origin (MEC-1 (Stacchini et al., 1999) and HG-3 (Rosén et al., 2012)) and the two other (MAVER-1 (Zamò et al., 2006) and MINO (Lai et al., 2002)) were originated from MCL. The cell lines differ not only in their origin but also in their capacity to activate BCR signaling, which was triggered only in MAVER-1 and MINO cell lines but not in the tested CLL lines (Supplementary Figure S1). The confinement caused a dramatic change in the phenotype of the studied cells (Figure 1B). MEC-1 cells showed highly polarized morphology with pronounced uropods and actively migrated (Supplementary Movie S1) and resembled a phenotype that we observed earlier in this cell line upon fibronectin coating (Kaucká et al., 2015). A similar morphology was observed in HG-3; however, in contrast to MEC-1, the morphology of this cell line was more variable, and the cells often formed clusters (Supplementary Movie S2). In contrast, the two MCL cell lines showed limited migratory properties and only rarely established the polarized migratory phenotype. Both MAVER-1 and MINO, however, showed highly dynamic protrusive activity, causing them to oscillate around the same spot (Supplementary Movies S3, S4).

### Quantitative comparison of the migratory properties of chronic lymphocytic leukemia and mantle cell lymphoma cell lines

**FIGURE 2:**
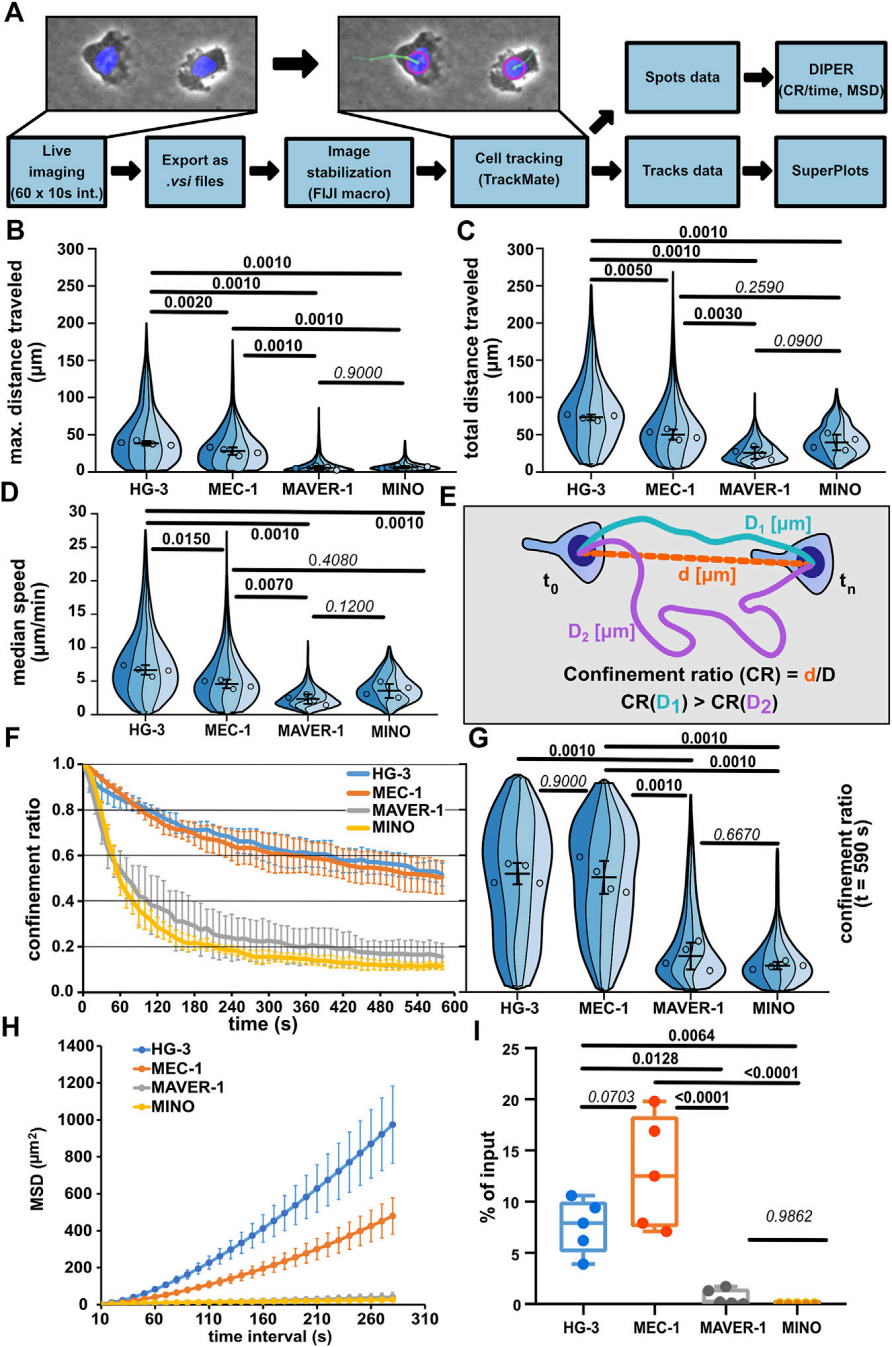
Comparison of migratory properties of leukemic cell lines. **(A)** Scheme of the data processing pipeline used for the quantification of migration parameters. FIJI macro used for pre-processing images for tracking can be found in Supplementary Materials. **(B)** Maximum distance traveled by the tested cell lines. SuperPlot sections of different colors represent distributions of individual replicates. Circles represent the median values of replicates, which were used for statistical analysis (one-way ANOVA, *n* = 4, *p* < 0.001, and the post hoc Tukey test) (*p* values shown in the graph). Error bar: mean and SD. **(C)** Total distance traveled by the tested cell lines. SuperPlots set as in Figure 2B. Statistics: one-way ANOVA, *n* = 4, *p* < 0.001, and the post hoc Tukey test. **(D)** Median speed of the tested cell lines. Superplot setting as in Figure 2B, statistics: one-way ANOVA, n = 4, *p* < 0.001, and the post hoc Tukey test. **(E)** Schematic depiction of the calculation of the confinement ratio (CR). D = length of the track (equals to “total distance traveled”) and d = cell displacement (direct distance from the first to the last point in the given track). **(F)** Evolution of the CR of the tested cell lines over the period of tracking. The lines represent average value calculated from the replicate medians; error bar: SD. **(G)** Comparison of the CR of the tested cell lines at the last frame of the tracking period (t = 590 s). SuperPlot settings are as in Figure 2B. Statistics: one-way ANOVA, *n* = 4, *p* < 0.001, and the post hoc Tukey test. **(H)** Mean square displacement (MSD) analysis of the tested cell lines. MSD shows an increase in the area explored by migratory cells over different time intervals. Line represents the average value of replicate MSD values calculated by DIPER; error bar: SD. **(I)** Transwell assay analysis of basal migration of the tested cell lines. Percentage of cells that migrated to the lower well of the chamber after 3 h incubation without chemokine gradient stimulation and normalized to the total number of cells pipetted to each well. Statistics: one-way ANOVA, *n* = 5, *p* < 0.001, and the post hoc Tukey test. The data presented in B, C, D, F, G, and H were derived from the same tracking experiments. Number of cells (tracks) used for quantification: HG-3 (76, 99, 193, 88), MEC-1 (149, 175, 387, 134), MAVER-1 (143, 95, 187, 87), and MINO (176, 99, 259, 172).

As the first step, we have decided to quantitatively describe the migratory parameters of the selected cell lines HG-3, MEC-1, MAVER-1, and MINO under confinement. The cells were stained with Hoechst 33342 and imaged under the agarose with a widefield microscope using a 10x objective in phase contrast and a blue fluorescence channel to image Hoechst-positive nuclei. The cells were tracked for 10 min at a frequency of 1 frame per 10 s for a total of 60 frames. The acquired time-lapse data were subsequently processed as schematized in Figure 2A and directly illustrated in Supplementary Movie S5. After the image data pre-processing, the cells were tracked based on the blue fluorescence channel using FIJI TrackMate plugin (v7.5.1), and only complete trajectories (containing all 60 spots/timepoints) were further analyzed. The migration parameters were used as calculated by TrackMate and the DIPER MS Excel macro (Gorelik and Gautreau, 2014) (for details, see Materials and methods).

Automatic cell tracking (Figures 2B, C) demonstrated that the CLL cell lines MEC-1 and HG-3 are highly migratory under confinement, even without any external stimulation. On the contrary, cell lines of MCL origin—MAVER-1 and MINO—were mostly nonmotile. Interestingly, HG-3 cells also on average migrated significantly more than MEC-1 cells. The differences between motile and nonmotile cell lines could be well distinguished using the “*max. distance traveled*” parameter (Figure 2B), which measures the maximal distance between any two timepoints in individual tracks. Of note, based on the “*total distance traveled*” parameter (Figure 2C), which measures the total length of cell trajectory and the median speed of migration (Figure 2D), MCL cell lines MAVER-1 and MINO appear more migratory. In the speed parameter, there appears to be no significant difference between MINO and MEC-1 cells, which is surprising given the obvious differences in their motility. This discrepancy is likely due to the aforementioned continuous protrusive activity in these nonmotile cells, which results in the oscillation of the nucleus and thus increases the overall track length (this phenomenon is demonstrated in Supplementary Movie S6, showing oscillatory MINO cells). This also affects the measurement of migration persistence by “*confinement ratio*” (CR), as this parameter is mathematically derived from the track length (Figure 2E). However, as the oscillatory cells do not move away from their original spot, the CR decreases. Consequently, the CR in both MAVER-1 and MINO cell lines is lower than that in migratory HG-3 and MEC-1 cell lines (Figures 2F, G). To highlight the differences between the motility of these cell lines, we have analyzed the “*mean square displacement*” (MSD) as a measure of the area explored by the cells during migration. MSD values clearly showed the higher migratory capacity of HG-3 and MEC-1 cells than that of MAVER-1 and MINO (Figure 2H). In light of these observations, we recommend using “maximum distance traveled” and MSD rather than track length or speed for a more accurate assessment of the real migratory potential of a given cell line.

When we quantified the migration of the four cell lines using the transwell assay (Figure 2I), we could very well recapitulate the key findings obtained by the under-agarose imaging. Under non-stimulated condition, the tested CLL lines HG-3 and MEC-1 show dramatically higher levels of motility compared to the tested MCL cell lines. This method of comparison suggests that the microscopic analysis of cell migration in the confined environment can help to mechanistically explain the quantitative changes discovered previously by transwell assays.

### CK1 inhibition disrupts chronic lymphocytic leukemia cell line migration differently to ROCK inhibition

To test the potential of this experimental system, we have decided to compare the migratory parameters of CLL cell lines upon treatment with previously reported inhibitors of the amoeboid migration of CLL cells. We have shown earlier using the transwell assay (Kaucká et al., 2013) that the migration of CLL cells can be inhibited both by inhibitors of casein kinase 1 (CK1) and by inhibitors of the RhoA–Rho-associated kinase (ROCK) axis. Our aim was to use direct tracking to identify inhibitor-specific changes in migratory parameters that would be missed in endpoint migration assays. For this experiment, we used two different inhibitors of CK1: the best-defined commercial CK1 inhibitor, PF670462 (Badura et al., 2007), and a novel inhibitor, MU1742, highly specific to CK1 isoforms δ and ε. As a reference, we decide to target ROCK because of its crucial importance for amoeboid migration in driving actomyosin contractility (Graziani et al., 2022). Furthermore, ROCK has been reported as one of the downstream effectors of non-canonical Wnt signaling (Carmona-Fontaine et al., 2008; LaMonica et al., 2009; Rodriguez-Hernandez et al., 2020). We took advantage of the widely used ROCK inhibitor Y27632 (Uehata et al., 1997), effects of which on migration and phenotype have been well described across lymphoid and other amoeboid cellular models (Bardi et al., 2003; Lee et al., 2004; Samaniego et al., 2007; Heasman et al., 2010; Takesono et al., 2010).

**FIGURE 3:**
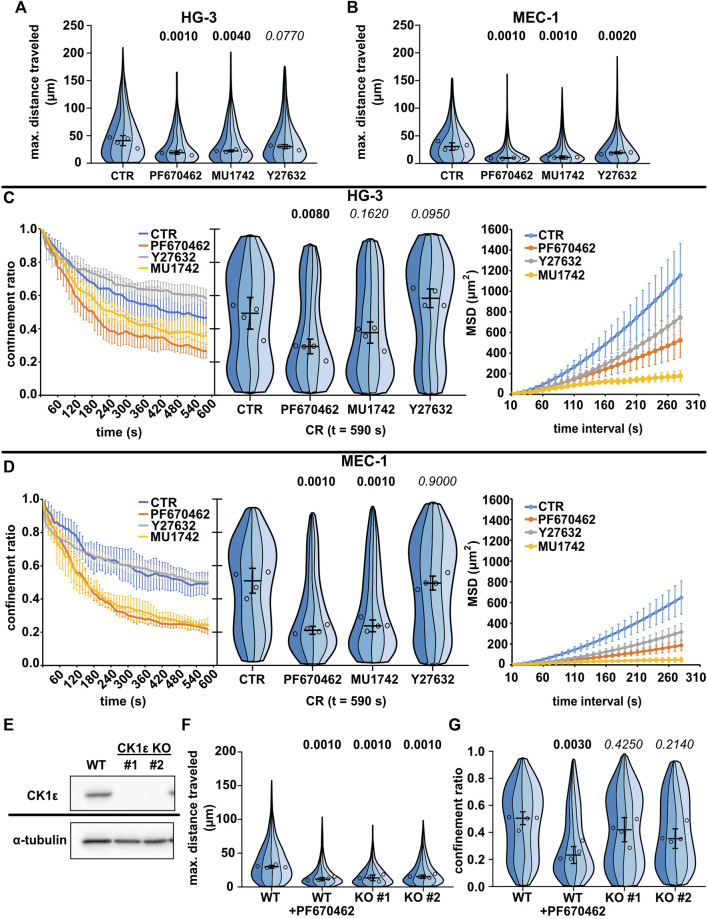
Effects of CK1 inhibitors PF670462 and MU1742 and a ROCK inhibitor Y27632 on the basal migratory properties of the CLL cell lines. **(A)** Maximum distance traveled by HG-3 cells treated with 10 μM of one of the indicated inhibitors or an equal volume of DMSO (CTR). Statistics: one-way ANOVA, n = 4, *p* < 0.001, and the post hoc Tukey test (*p* values of comparison to CTR condition). **(B)** Maximum distance traveled by MEC-1 cells in the same experimental setup. Statistics: one-way ANOVA, n = 4, *p* < 0.001, and the post hoc Tukey test). **(C)** Analysis of HG-3 migratory persistence under the effect of CK1 and ROCK inhibitors. Left: Decay of CR over the period of tracking. Center: CR values at the last frame of the tracking period (t = 590 s). Statistics: one-way ANOVA, n = 4, *p* < 0.001, and the post hoc Tukey test. Right: MSD analysis. **(D)** Analysis of MEC-1 migratory persistence under the effects of CK1 and ROCK inhibitors. Left: decay of CR over the period of tracking. Center: CR values at the last frame of the tracking period (t = 590 s). Statistics: one-way ANOVA, n = 4, *p* < 0.001, and the post hoc Tukey test. Right: MSD analysis. **(E)** Western blot validation of the loss of expression following the CRISPR/Cas9 knockout of CK1ε in the MEC-1 cell line. α-Tubulin signal is shown as a loading control. Representative images from three biological replicates. **(F)** Comparison of migratory properties of two MEC-1 CK1ε KO clones to the unmutated culture (WT) using the maximum distance traveled parameter. Statistics: one-way ANOVA, *n* = 4, *p* < 0.001, and the post hoc Tukey test. **(G)** Comparison of the confinement ratio parameter between the CK1ε KO clones and the unmutated cell line from the same experiment. Statistics: one-way ANOVA, *p* = 0.004, and the post hoc Tukey test. SuperPlot setting in all panels is consistent with that described in Figure 2B. Number of cells (tracks) used for quantification in **(A** and **C)** (same experiment): CTR (103, 259, 89, 100), PF670462 (80, 77, 73, 162), MU1742 (111, 103, 166, 131), and Y27632 (103, 259, 89, 100). Number of cells (tracks) quantified in **(B** and **D)** (same experiment): CTR (141, 98, 84, 38), PF670462 (182, 87, 232, 82), MU1742 (105, 155, 166, 180), and Y27632 (115, 144, 192, 160). Number of cells (tracks quantified in **(F-G)** (same experiment: WT (292, 219, 399, 205), WT + PF670462 (228, 138, 239, 271), KO#1 (126, 162, 90, 299), and KO#2 (159, 229, 149, 110).

We tested the effects of these inhibitors first in the two spontaneously motile cell lines (MEC-1 and HG-3) (Figure 3, Supplementary Movies S7–S12). As expected, both CK1 inhibitors reduced the migration of both CLL cell lines at the level of maximum distance traveled (Figures 3A, B) and at the level of mean square displacement (Figures 3C, D). We have also checked the effects on total track length, which show similar trends (Supplementary Figure S2A,B). Interestingly, the two cell lines differed in their response to Y27632; while MEC-1 cells were inhibited in their migration as expected, the effect was not statistically significant in HG-3 cells. Strikingly, however, the inhibitors differed in their effect on migration persistence, while the treatment with both CK1 inhibitors significantly decreased the confinement ratio (in the HG-3 cell line only with the PF670462 inhibitor), this parameter did not change upon treatment with the ROCK inhibitor (Figures 3C, D). Combined treatment with both inhibitors (Supplementary Figure S2C,D) revealed that the effect of CK1 inhibition is independent on ROCK inhibition in MEC-1 cells. We conducted the same experiments also for the two MCL cell lines (Supplementary Figure S3), but due to their low basal motility, we were not able to observe any clear effects apart from the decrease of MSD upon CK1 inhibition in the MINO cell line (Supplementary Figure S3F).

As an alternative approach to verify the importance of CK1 for the migration of leukemic B cells, we have tested the migratory properties of MEC-1 cells where the gene encoding for CK1ε was disrupted by CRISPR/Cas9-mediated gene editing. Two CK1ε CRISPR/Cas9 knockout clones of the MEC-1 cell line have been tested. The loss of CK1ε expression in these clones has been verified by western blot (Figure 3E). Importantly, both clones have shown decreased migratory abilities than the control wild-type cells (Figure 3F). The effect, however, was not as strong as that observed for the biochemical inhibition of CK1 by PF670462 (Figure 3G), likely due to the partial redundancy of CK1ε and CK1δ. Altogether, this analysis provides genetic proof for the importance of CK1ε in amoeboid cell migration and validates the results obtained with CK1 inhibitors.

More detailed analysis of the cell morphology of cells in the confined environment uncovered further differences. Y27632 in both HG-3 and MEC-1 cell lines caused elongated adherent phenotypes, likely resulting from the defects of trailing edge (uropod) de-adhesion observed previously in T lymphocytes (Smith et al., 2003). In some cells, the same inhibitor caused the formation of curious axon-like protrusions with moving lamellipodium at their ends (Supplementary Movies S9, S12). On the contrary, the cells treated with PF670462 lacked any striking morphological alteration. However, on average, they showed a less polarized phenotype (Supplementary Movies S8, S11).

**FIGURE 4:**
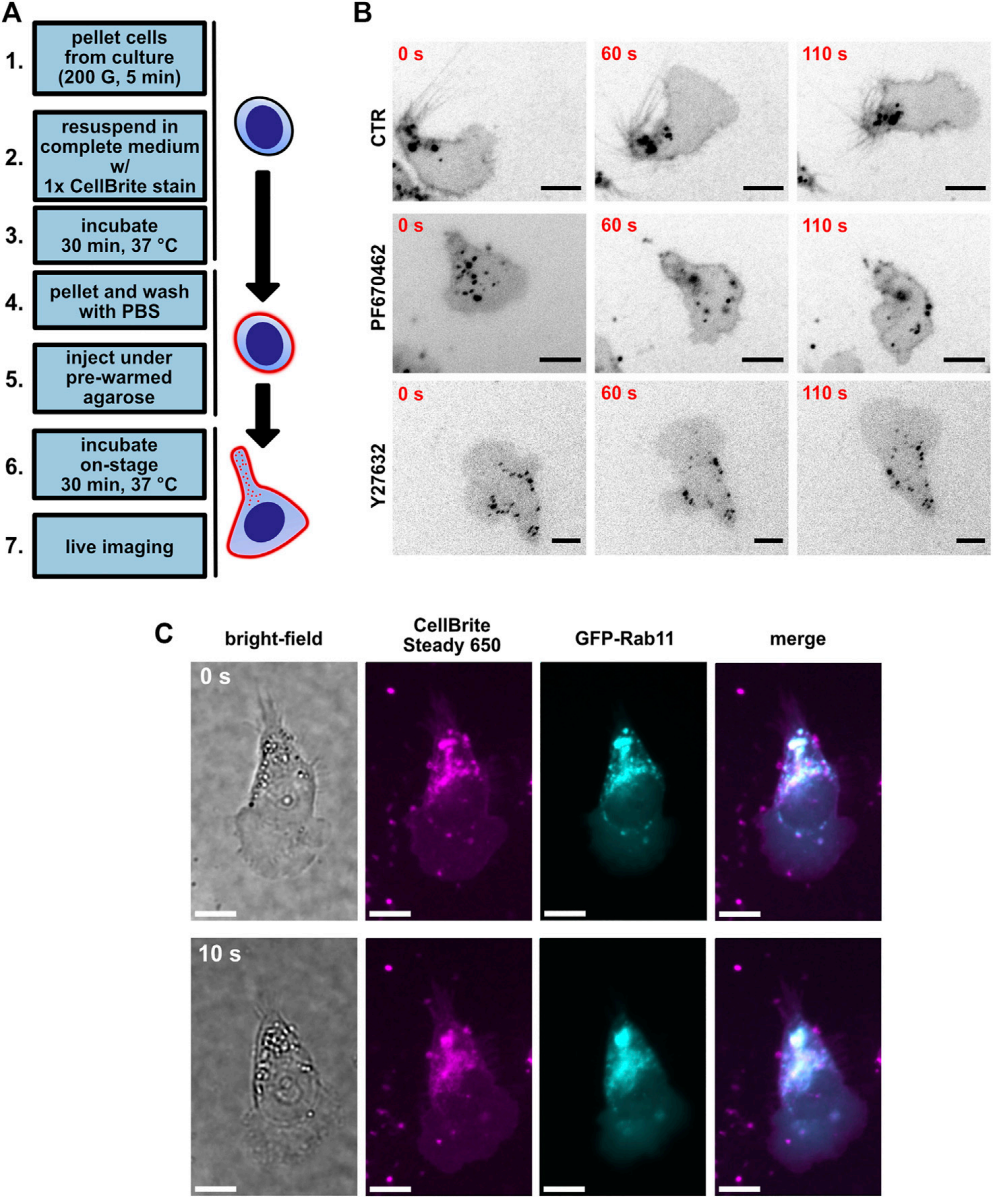
Visualization of uropod endocytic polarity using the CellBrite plasma membrane stain. **(A)** A scheme of the protocol used for plasma membrane staining. **(B)** MEC-1 cells stained with CellBrite Steady 650 plasma membrane stain (black); scale bar: 10 µm. Shown are three timepoints from a time-lapse experiment. Upper lane: control condition treated with DMSO. Middle: MEC-1 cells treated with 10 µM PF670462. Bottom: MEC-1 cells treated with 10 µM Y27632. **(C)** Representative image of GFP-Rab11 overexpression experiment in MEC-1 cells. Shown are two timepoints from a time-lapse experiment. Magenta: CellBrite Steady 650. Cyan: GFP-Rab11. Scale bar: 10 µm.

To gain further insight into the morphological changes of MEC-1 cells, we decided to use a live membrane fluorescence stain. It has been shown earlier in T cells that the polarized distribution of endocytic vesicles to the uropod represents a very useful readout that brings an important insight into the polarity of the lymphocytes (Samaniego et al., 2007). Indeed, live visualization of membrane vesicles by CellBrite plasma membrane stain (for schematics, see Figure 4A, and for time-lapse videos of these experiments, see Supplementary Movie S13) showed their clear accumulation in the uropod of MEC-1 cells (Figure 4B, upper row) that closely resembled the situation described in T cells (Samaniego et al., 2007). To verify that the punctate structures visualized by CellBrite are indeed endocytic vesicles, we have co-transfected MEC-1 cells with a plasmid encoding GFP-tagged Rab11, the well-characterized marker of this type of vesicle (Wilcke et al., 2000). As shown in Figure 4C, Rab11 co-localized with the CellBrite signal, which provides proof that even in B cells, endocytic vesicles localize into the uropod as in T cells (Biberfeld, 1971; Samaniego et al., 2007). The distribution of the vesicles appeared more diffuse after treatment with either PF670462 (Figure 4B, middle) or Y27632 (Figure 4B, bottom). Subsequently, we have attempted to quantitatively describe the distribution pattern of the vesicles (Supplementary Figure S4). We have developed two analytical pipelines that use the median distance between the vesicles as the key parameter. The hypothesis is that this parameter will decrease when the vesicles accumulate in one part of the cell and increase when they distribute evenly. The pipelines described in further detail in Supplementary Figure S4 and Methods section detected a statistically significant difference in the case of Y27632-treated cells but failed to detect the difference between control and PF670462-treated cells. It remains to be resolved if this is due to the low sensitivity/inherent limitations of these analytical approaches or to a lack of biological difference. The raw image datasets will be made freely accessible via an online repository to facilitate further analyses by other researchers (see Data Availability Statement section).

Altogether, data presented in Figures 3 and 4 show that both CK1 and ROCK inhibition disrupt the migratory polarity of CLL cells, despite showing different morphological and migratory outcomes. While the role of ROCK in uropod endocytic trafficking and migratory polarity has been studied previously on T-cell models (Smith et al., 2003; Lee et al., 2004; Samaniego et al., 2007; Takesono et al., 2010), the upstream polarity pathways (including CK1 function) acting at lymphocyte polarity remain to be elucidated despite their emerging importance (Ludford-Menting et al., 2005; Zyss et al., 2011; Kaucká et al., 2015). Our model provides a useful and robust functional assay to address these questions.

### Under-agarose assay allows study of the chemokine and VCAM1 stimulation

**FIGURE 5:**
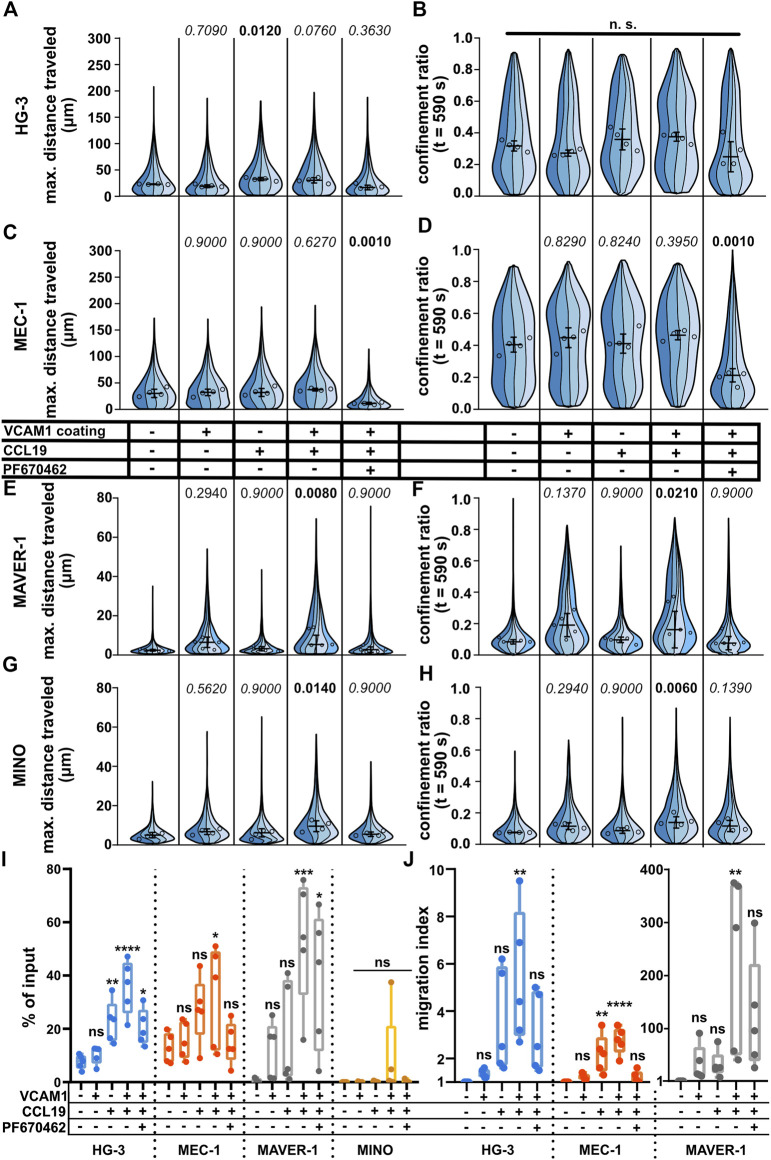
Effects of positive stimulation by coating or chemokines on the migration of the tested cell lines. The cells were stimulated by the well surface coated with 2 μg/ml VCAM1 and/or treatment with CCL19 (200 ng/ml) and/or 10 μM. See the condition legend in the center of the figure. Due to the confounding effects of oscillating cells on “total distance traveled,” which are described in detail in the main text, we decided to focus mainly on “maximum distance traveled” instead to describe the effects of stimulation more accurately. **(A)** Maximum distance traveled by HG-3 cells. Statistics: one-way ANOVA, *n* = 4, ***p*** < 0.001, and the post hoc Tukey test (*p* values of comparison to unstimulated condition). **(B)** CR at the last frame of the tracking (t = 590 s) of the HG-3 cell line (one-way ANOVA, n = 4, and *p* = 0.090) **(C)** Maximum distance traveled by MEC-1 cells. Statistics (one-way ANOVA, n = 4, ***p*** < 0.001, and the post hoc Tukey test). **(D)** CR at the last frame of the tracking (t = 590 s) of the MEC-1 cell line (one-way ANOVA, n = 4, *p* < 0.001, and post hoc Tukey test). **(E)** Maximum distance traveled by the MAVER-1 cell line (one-way ANOVA, n = 5**,** *p* = 0.005**,** and the post hoc Tukey test). **(F)** CR at the last frame of the tracking (t = 590 s) of the MAVER-1 cell line (one-way ANOVA, n = 5, *p* = 0.005, and the post hoc Tukey test). **(G)** Max. distance traveled by the MINO cell line (one-way ANOVA, *n* = 4, *p* = 0.018, and the post hoc Tukey test). **(H)** CR at the last frame of the tracking (t = 590 s) of the MINO cell line (one-way ANOVA, n = 4, *p* = 0.006, and the post hoc Tukey test). SuperPlot settings in all panels are consistent with those described in Figure 2B. Numbers of cells (tracks) quantified in **(A–B)** (same experiment), left to right: 144, 311, 72, 157; 151, 169, 157, 169; 158, 222, 135, 100; 91, 195, 143, 117; 180, 230, 275, and 236. **(C–D)** (same experiment), left to right: 210, 248, 163, 121; 223, 259, 258, 317; 184, 246, 95, 144; 184, 200, 208, 251; 176, 305, 347, and 101. **(E–F)** (same experiment), left to right: 92, 293, 167, 237, 129; 210, 334, 128, 233, 118; 275, 604, 241, 153, 56; 194, 325, 182, 173, 89; 191, 173, 188, 111, and 75. **(G–H)** (same experiment), left to right: 88, 255, 164, 142; 187, 68, 203, 114; 162, 266, 195, 178; 222, 185, 197, 157; 347, 273, 192, and 188. **(I)** Transwell assay analysis of positive stimulation and effect of CK1 inhibitor on migration of the tested cell lines. Percentage of the cells that migrated to the lower well of the chamber after 3 h of incubation normalized to the total number of cells loaded per well. Statistics: one-way ANOVA (*n* = 5) calculated for individual cell lines. HG-3: *p* < 0.0001, the post hoc Tukey test of comparison to the unstimulated condition, and *p* values from left to right: 0.9236, 0.0077, <0.0001, and 0.0245. MEC-1: *p* = 0.05 and the post hoc Tukey test *p* values: 0.9856, 0.1772, 0.0475, and 0.9974. MAVER-1: *p* = 0.0016 and the post hoc Tukey test *p* values: 0.7110, 0.4464, 0.0008, and 0.0155. MINO: *p* = 0.2908. **(J)** Transwell migration data from the same experiment as panel **(I)** expressed as the migration index (normalized to the untreated control). Data from MAVER-1 were plotted separately due to the large difference to HG-3 and MEC-1 cell lines. The migration index for the MINO cell line could not be calculated due to the low numbers of transmigrated cells among the experimental conditions. Statistics: one-way ANOVA (n = 5) calculated for individual cell lines. HG-3: *p* = 0.0062, the post hoc Tukey test of comparison to an unstimulated control, and *p* values from left to right: 0.9925, 0.1065, 0.0033, and 0.2237. MEC-1: *p* < 0.0001 and the post hoc Tukey test *p* values: 0.8776, 0.0031, <0.0001, and 0.9836. MAVER-1: *p* = 0.0049 and the post hoc Tukey test *p* values: 0.9417, 0.9506, 0.0029, and 0.1365.

Under (patho)physiological conditions, lymphocyte migration is regulated by multiple stimuli. Among the most important is the migration toward the source of chemokine(s) (chemotaxis) or the interaction of cellular integrins with the components of the extracellular matrix and other cells. Even though the under-agarose assay allows for the formation of the chemotactic gradient for directional stimulation (Kopf et al., 2020), we decided to use a uniform concentration of chemokines to make sure that all cells were stimulated with the same dose of chemokine. Since chemokines often act in synergy with integrins (Montresor et al., 2012), we also decided to check whether the migration could be further enhanced by coating the dish with vascular cell adhesion molecule (VCAM1), a molecule complementary to the abundant B-lymphocyte integrin VLA4 (Härzschel et al., 2020). As shown in Figures 5A–H, we could clearly quantify a positive migratory response to both chemokine CCL19 (200 ng/ml) and VCAM1 treatments. Interestingly, each of the tested cell lines reacted in a different pattern. While MEC-1 cells reacted to neither chemokine nor integrin stimuli, HG-3 showed a significant increase in migration upon treatment with CCL19, however, without the additive effect of VCAM1. Contrarily, MAVER-1 and MINO cell lines responded significantly only to the co-stimulation by CCL19 and VCAM1. The response was most striking in MAVER-1 cells, which started to migrate upon CCL19/VCAM1 co-stimulation, and this effect is clearly visible by eye (compare Supplementary Movies S14, S15). Finally, CK1 inhibition efficiently attenuated this positive effect of CCL19 + VCAM1. We have also tested the effects of CXCL12 (SDF1a) chemokine in the same setup (Supplementary Figure S5A–H), which showed similar activity patterns among the tested cell lines. While neither VCAM1 coating nor CXCL12 stimulation stimulated response in HG-3 and MEC-1 cells, it increased migration of MAVER-1 cells when the stimuli were combined. The response of MINO cells appeared to be dependent mainly on the VCAM1 coating rather than on chemokine stimulation. The observed changes cannot be attributed to direct effects of chemokine treatment on CK1ε protein levels, as validated by western blotting (Supplementary Figure S5I).

For comparison of methods, we also repeated the CCL19 stimulation using the transwell assay. The results are shown both as the percentage of input (Figure 5I) and as the migration index (MI) which reflects fold change in comparison to the untreated control (Figure 5J). In general, the results showed similar trends to cell tracking experiments with some remarkable differences. Both HG-3 and MEC-1 cells increased their migration upon stimulation with CCL19, regardless of the VCAM1 coating. MAVER-1 cells responded very strongly to both CCL19 and VCAM1 and even more to their combination with MI > 100. Interestingly, this boost of migration was only partially inhibited by CK1 inhibition in the transwell assay (Figure 5I) in comparison to the clear effects of the same treatment in the under-agarose assay (Figures 5E, F). We were not able to quantify any changes in the MINO cell line, as there were few transmigrated cells in all experimental conditions. This could be likely attributed to the fact that MINO cells are nearly triploid (Lai et al., 2002) and as such cannot pass the 5-µm pores used in this experiment efficiently due to their larger nuclei.

Our methodology determined clear differences in the migratory features of the tested CLL and MCL cell lines. To address whether our pipeline can be used in the analysis of primary CLL cells, we have collected primary CLL cells from six patients and tested their migration upon positive stimulation with CCL19/CXCL12 chemokines and VCAM1 coating. As shown in Supplementary Figure S6, the majority of the patients did not respond to the treatment. However, in some cases (most clearly in patient #5) we have observed a clear stimulation with both chemokines, VCAM1, and their combination. These results suggest that our experimental system and analytical pipeline can be used in the analysis of primary patient material and can help with functional patient stratification.

### Multiparametric analysis of integrin and chemokine receptor expression reveals differences in cell line phenotype homogeneity

**FIGURE 6:**
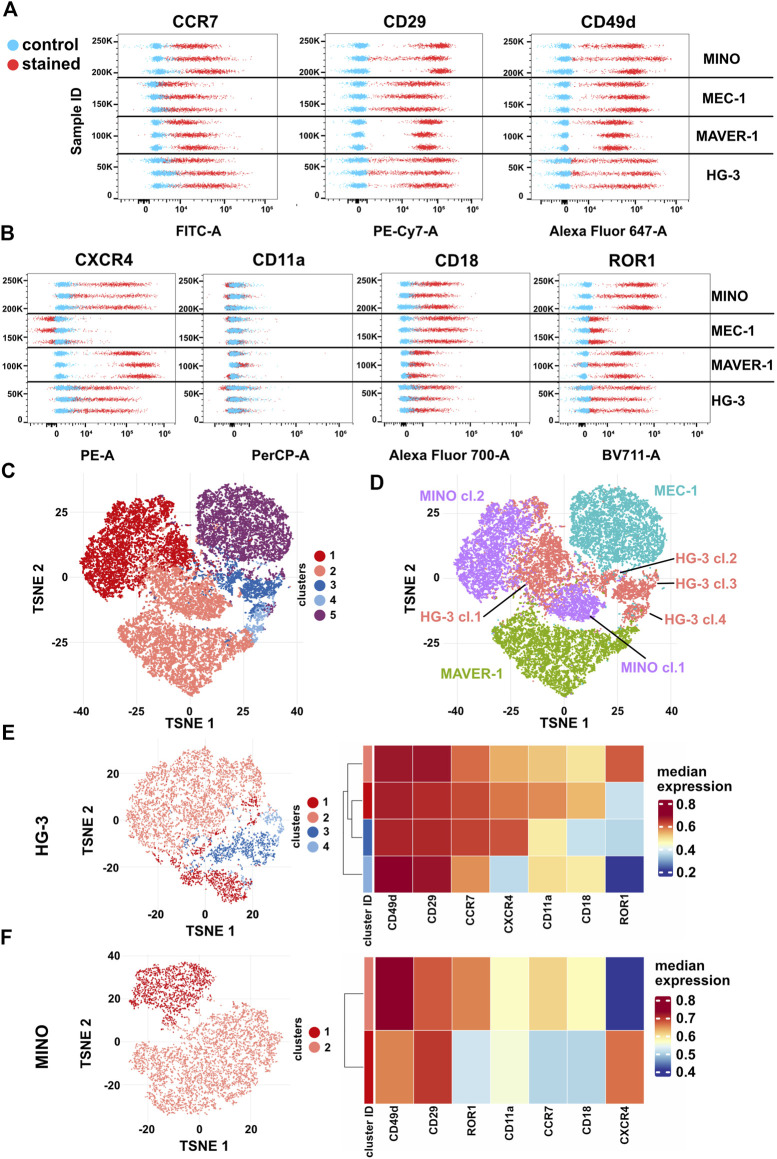
Expression of surface markers **(A)** CCR7 (CCL19 receptor), CD29 and CD49d (VLA4 integrin subunits), **(B)** ROR1 and CXCR4 (CXCL12 receptor), and CD11a and CD18 (LFA1 integrin subunits) in HG-3, MAVER-1, MEC-1, and MINO cells. Stained (red) and unstained control (blue) samples are shown as dot plots of fluorescence intensity. Three replicate measurements for each cell line are shown; for quantification, see Supplementary Figure S5. **(C)** Representative tSNE visualization of metaclusters automatically generated by FlowSOM of HG-3, MAVER-1, MEC-1, and MINO cells from one replicate shown in **(A–B)**. **(D)** tSNE visualization of cell line distribution. **(E)** Left: tSNE of the HG-3 cell line upon subtraction from the dataset shown in 5C. Right: heatmap resuming expression of individual markers in subclusters of the HG-3 cell line. **(F)** Left: tSNE of the MINO cell line upon subtraction from the dataset shown in 5C. Right: heatmap resuming expression of individual markers in subclusters of the MINO cell line.

Finally, we have decided to correlate the differences in migratory behavior of individual cell lines with the presence of CCL19 and VCAM1 receptors. The abundance of CCR7 (the receptor of CCL19) and subunits CD29 (integrin β1) and CD49d (integrin α4), which together form VLA4 integrin specific for VCAM1, in these cell lines was analyzed by spectral flow cytometry (Figure 6A, Supplementary Figures S7A–C). All cell lines were positive for CCR7, CD29, and CD49d; interestingly, we have not observed a direct correlation between the amount of the receptors and the physiological response to the ligands. However, we have observed signs of heterogeneity intrinsic to individual cell lines, which were most obvious in HG-3 and MINO. This motivated us to test the expression of four additional membrane markers associated with cell migration: CXCR4 (chemokine receptor), ROR1 (Wnt5A receptor), and CD11a/CD18 (αL and β2 subunits forming LFA1 integrin) (Figure 6B, Supplementary Figures S7D–G). The surface levels of any of these receptors have not been affected by the inhibitors used in this study (Supplementary Figure S8).

Based on these seven markers, individual single cells from all four tested cell lines were analyzed and visualized as a tSNE plot with metaclusters automatically identified using FlowSOM (Quintelier et al., 2021) (Figure 6C). Interestingly, this analysis clearly revealed not only (expected) differences among individual cell lines but also remarkable variability within the HG-3 (four distinct clusters) and MINO (two distinct clusters) subpopulations (Figure 5D). Detailed analysis of individual clusters (Figures 6E, F) showed that in HG-3, the variability arises mainly from ROR1, CXCR4, CD11a, and CD18 (thus LFA1) surface expression, whereas MINO subpopulations differ in more markers, including both screened chemokine receptors, CD18 and ROR1. We believe that such diversity in the expression of key regulators of migration and chemotaxis brings additional variability to the assays presented in this study, and we propose that flow cytometric detection and quality control shall be implemented as a routine check to allow higher reproducibility between cell lines and research teams. As a proof of this concept, we used FACS to sort the two most distinct subpopulations of the HG-3 cell line based on their ROR1 expression and then compared their migratory properties. After sorting, the ROR1^+^ and ROR1^-^ subclones maintained their ROR1 expression phenotype for at least several days, which we verified through routine flow cytometry testing (Supplementary Figure S9). Interestingly, we were indeed able to identify a significant difference in migratory properties between these subclones, even though they did not differ significantly from the unsorted culture (Supplementary Figures S9B–D). Overall, the ROR1^+^ cells showed slightly lower migration capabilities than ROR1^-^ as measured by both maximum and total distance traveled and a decreased confinement ratio.

## Discussion

In this study, we have summarized our observations of the migratory behavior of four cancer cell lines originating from the B-cell lineage: HG-3, MEC-1, MAVER-1, and MINO. Using a modification of the long-established under-agarose assay (Nelson et al., 1975; Hons et al., 2018) together with live imaging microscopy and open-source image analysis software (Schindelin et al., 2012; Tinevez et al., 2017), we have established a simple and reproducible pipeline for quantification of amoeboid migration of B-cell leukemic and lymphoma cell types. We believe that spatial confinement might also be a more relevant model than simple 2D migration, as it at least on a mechanical level reconstructs the environment the leukemic cells experience in lymphoid organs, where they accumulate and where the main physiological parallel of their behavior (migration of centroblasts in the germinal center) takes place (García-Muñoz et al., 2012).

Despite the fact that our pipeline is robust, we could identify some pitfalls that might conceal the real differences between the behaviors of the cells if not reviewed critically. This mainly refers to the artifacts in the confinement ratio (also known as the directionality ratio), which have been previously summarized by Gorelik and Gautreau (2014). Our results, however, show a novel phenomenon for this and other parameters including the measurement of migration speed arising from the tracking of cell nuclei in non-migratory cells with high protrusive activity. This results in an apparent increase in total track length and a subsequent decrease in CR. This effect should be accounted for in similar experiments before making conclusions about migratory persistence. This is perhaps best illustrated in the MAVER-1 cell line (Supplementary Figures S3A–B), where the values of the total distance in condition do not contain values bordering on 0, which is in striking contrast with the values of the maximum distance. Consequently, we argue that the maximum distance traveled or MSD might in similar experiments provide a better description of the migratory abilities of the cells than the total length of the track or speed.

The comparison of our setup with the transwell assay upon both negative and positive migratory stimulation highlighted the importance of direct quantitative observation for new insight into the cell migration of the leukemic B cells. First, our experiments with CK1 and ROCK inhibitors identified a difference between their effects on cell migration. As this difference arises purely from the geometries of the registered migratory trajectories and not from the overall distance traveled, this effect would not be discovered using indirect methods. Second, while the transwell assay reproduced the results from the under-agarose assay in the basal migration comparison (Figure 2), significant differences could be observed upon the addition of positive stimuli (Figure 5). While the absence of a chemokine gradient in our experimental setup could also play a role in this matter, this experiment reveals that multiple factors need to be considered to avoid misinterpretation of the data from transwell assays. The discrepancy between lower effects of CK1 inhibition on cell migration in transwell compared to our assay can be simply a question of the migrated distance. As the polycarbonate transwell membrane is only around 10 µm thick, we can speculate that even with impaired migratory abilities, the cells can upon directional stimulation easily cross this distance. As in the direct setup, we follow the cells over hundreds of micrometers, making this approach arguably much more sensitive. In Figure 6, we have shown that there are distinct subpopulations differing in the chemokine receptor and integrin expression even in the established cell lines. We have also shown that these subpopulations could differ even in their basal migration (Supplementary Figure S9). It is not bold to assume that these subpopulations will respond differently to chemotactic stimuli. In such a case, transwell would act as a filter, passing through only responding cells; thus, the number of transmigrated cells would just mirror their representation in the culture. By measuring migration directly, we observe the whole population of the cells in culture, represented by the shape of the SuperPlot (Lord et al., 2020; Kenny and Schoen, 2021), and its changes upon treatment. Finally, differences in cell sizes cannot be omitted in the transwell experimental design, complicating mutual comparison when different pore sizes need to be applied. This problem also does not arise with direct tracking.

The assay still allows for further development. One of the promising directions to follow in the future is the quantitative analysis of cells with stained membranes. CellBrite staining appears to be a useful tool for the analysis of molecular mechanisms that control the establishment, maintenance, and positioning of the uropod. Tracking of individual endocytic vesicles is possible and can be performed in the context of a whole migrating cell. Our results suggest that the assay allows for automatized quantification (Supplementary Figure S4), which will be developed in the near future.

Our data show that cell lines of similar origin show striking differences in their migration. Interestingly, the migratory behavior of the studied B cells is not always connected to chemotaxis. This is illustrated by the differences between MEC-1 and MAVER-1 cells: MEC-1 is highly migratory but does not react to chemotactic or integrin stimuli in the under-agarose assay. In contrast, MAVER-1 appears to be a good chemotaxis model, as the cells require a specific combination of stimuli to start to migrate and express a multifold increase in activity following the stimulus as observed by both under-agarose confinement and the transwell assay. We would also advise using these two cell lines given their relative homogeneity in the key migratory receptors. The flow cytometry analysis we performed as a quality check has revealed that the HG-3 and MINO cell lines contain several subpopulations that differ significantly from each other in their chemokine receptor and integrin expression. This is especially evident for the HG-3 cell line, which shows high internal heterogeneity, which might complicate the interpretation of experimental results based on this cell line.

One of the aims of this study was to observe how CK1 inhibition affects the ability of these cell lines to migrate and respond to stimuli. Indeed, treatment with CK1 inhibitors or knock-out of CK1ε (as demonstrated in MEC-1 cells) severely impaired the migratory abilities of all tested cell lines either by decreasing their basal motility or by attenuating their response to chemotactic stimuli. CK1 isoforms, CK1δ and CK1ε, are the important components of β-catenin-independent Wnt signaling pathways. Wnt5A, a ligand commonly associated with these pathways, is responsible for autocrine amplification of T-lymphocyte response to chemokine stimulation (Ghosh et al., 2009) and is responsible for enhanced migratory properties of leukemic T cells in adult T-lymphocytic leukemia (Ma et al., 2013; Deng et al., 2017; Nakano et al., 2021). Our previous studies have also shown that higher expression of Wnt5A is associated with deregulated chemotaxis in CLL patient samples (Janovska et al., 2016). The precise molecular mechanism leading from CK1 activity to the defects of cell migration has, however, not been fully identified yet. It is possible that CK1 regulates the establishment of cell polarity, which is necessary for persistent migration. Planar cell polarity (PCP) signaling is one of the established downstreams of Wnt signaling (Yang and Mlodzik, 2015); however, its role in amoeboid cell models is not yet understood (Čada and Bryja, 2021). We have previously shown that CK1 inhibition can disrupt the polarized distribution of overexpressed VANGL2 polarity protein in the MEC-1 cell line; however, the importance of this protein for immune cell migration is a matter for further research (Kaucká et al., 2015). Interestingly, CK1 inhibition has been shown to disrupt the reorientation of the centrosome toward the immunological synapse in T cells (Zyss et al., 2011), which strengthens the connection with PCP, which has been shown to regulate the orientation of related organelles in various other cellular models (Carvajal-Gonzalez et al., 2016). This hypothesis is strengthened by our observations on the MEC-1 cell line, which showed a prominent accumulation of endocytic vesicles in the uropod in untreated migratory cells, which is consistent with previous studies observing increased endocytosis in the uropod (Samaniego et al., 2007), a process associated with centrosome positioning in lymphocytes (Kupfer and Dennert, 1984; Ratner et al., 1997). Upon treatment with the CK1 inhibitor, this accumulation appeared disrupted, suggesting defects in stabilizing the centrosome and associated organelles such as the Golgi apparatus toward the uropod, resulting in less stable migratory polarity. We cannot rule out, however, that these changes occur as a more general result of a defective migratory polarity caused by CK1 inhibition and therefore might not be causative. We have attempted to quantify the observed phenotypes *via* the measurement of vesicle distribution based on mutual distances using two different approaches. We have, however, failed to recapitulate our observations this way. Nevertheless, we think that this type of analysis might not be suitable for the cell phenotypes we aim to describe. Given that lymphocytes are typical with their very high nucleus–cytoplasm ratio, in nonpolarized cells, there is only a very limited area for vesicles to fit in. Larger differences in vesicle distances could thus be attained only when larger protrusions are present, such as those occurring after ROCK inhibition, where we indeed identified a significant difference. Due to the previous reasons, we decided to present both results to the reader for evaluation.

### Concluding remarks

Our results provide an important reference point for further studies of the migration of normal and transformed B lymphocytes. We provide a proof-of-concept that an experimental pipeline can track changes induced by biologically relevant stimuli or by small molecules known to interfere with amoeboid cell migration. Admittedly, our protocol combines several previously published methods; nevertheless, we would like to argue that the significance of our approach is the successful integration of these parts (under-agarose assay, automatic image analysis, and data visualization) into a simple and robust pipeline applicable to a wide variety of migratory cells, exceeding the field of leukemia research. The usefulness of our experimental system is proven by the discovery of different modes of action of CK1 and ROCK1 inhibitors, a phenomenon that would be missed in the transwell assay. We hope that this work will help other researchers in the design and interpretation of their research on leukemias and lymphomas.

## Materials and methods

### Cell lines and culture

All cell lines used in this study were obtained from German Collection of Microorganisms and Cell Cultures (DSMZ). We have used two cell lines of CLL origin: MEC-1 (cat. no. ACC 497) and HG-3 (cat. no. ACC 765) and two cell lines of MCL origin: MAVER-1 (cat. no. ACC 717) and MINO (cat. no. ACC 687). Between experiments, the cell lines were cultured in an incubator (37°C, 5% CO_2_) in RPMI 1640 medium (Biosera, cat. no. LM-R1640/500) supplemented with 10% fetal bovine serum (Gibco) and 1% penicillin–streptomycin (Biosera), further referred to as “*complete RPMI medium*.”

### Patient samples

The peripheral blood of CLL patients was taken after written informed consent in accordance with the Declaration of Helsinki under protocols approved by the Ethical Committee of University Hospital Brno. B cells were isolated using gradient centrifugation coupled with the depletion of non-B cells (RosetteSep CD3^+^ Cell Depletion Cocktail, RosetteSep B Cell Enrichment Cocktail, StemCell Technologies). The separation efficiency was assessed by flow cytometry; all samples contained ≥98% B cells. After isolation, the cells were cultured using the same media and incubation environment as described earlier. Migration of the primary cells was analyzed approx. 24 h after isolation.

### Under-agarose migration experiments

For the migration experiments under agarose, we modified a protocol previously reported (Hons et al., 2018). The final agarose gel mixture resulted from two components: the first component was a medium consisting of 66% RPMI 1640 (sup. with 20% FBS) and 33% 2X HBSS (Gibco, cat. no. 14185-045), and the medium was preheated in a water bath up to 37°C. The second component was a solution of 2% agarose (TopVision Low Melting Point Agarose, Thermo Scientific, cat. no. R0801) in deionized water. The agarose was dissolved in the water using several repeated short heating periods in microwave. After the agarose solution became fully transparent, it was placed into the water bath, which had been pre-warmed to 37°C to equilibrate the temperatures of the components. After 10 min, the two components were rapidly mixed in a ratio of 3:1 (medium–agarose) and further incubated at 37°C to avoid solidification. During experiments with inhibitors, the mixture was then divided into smaller volumes to which the inhibitors or DMSO (in the control condition) were added. Both CK1 inhibitors, PF670462 (DC Chemicals) and MU1742, and ROCK inhibitor Y27632 (Merck) were used at a final concentration of 10 µM. The total volume of the inhibitor/DMSO added equaled 1:1,000 of the resulting mixture. Recombinant human CCL19 (RnD, cat. no. 361-MI) and CXCL12 (RnD, cat. no. 350-NS) chemokines were dissolved in 0.1% bovine serum albumin (BSA, Serva) in PBS. The final concentration of chemokine in the agarose gel was 200 ng/ml.

The resulting agarose mixture was poured into µ-Slide 8 Well ibiTreat (ibidi, cat. no. 80826) at 300 µl/well. In the case of coating experiments, the µ-Slides were pre-treated with 0.1% BSA in PBS with or without 200 ng/ml recombinant human VCAM1 (RnD, cat. n. 862-VC) overnight at 4 °C. The coating solution was completely removed directly before adding the agarose mixture. Upon filling the wells, the slides were placed into sterile plastic dishes with a piece of wet cotton to avoid drying. The dishes were sealed using parafilm and incubated for 1 h at 4°C. After this step, we used a sterile 2 mm UniCore punch (Qiagen) to make a hole in the now solid gels at one side of every well, which would later serve for the removal of the excessive medium. After that, the slides were placed into an incubator for 30 min for warming up and pH equilibration.

Approx. 500,000 cells were harvested from the culture and centrifuged (200 g, 5 min), and the pellets were resuspended in 200 µl complete RPMI medium supplemented with 10 μg/ml Hoechst 33342 (ThermoFisher) and incubated at room temperature for 10 min. After that, the cells were centrifuged again, the medium with Hoechst was removed, and the cell pellets were resuspended in 40 µl of a fresh complete RPMI medium. Then, 1 µl of the cell suspension was injected under the agarose into each well using a 10-µl pipette. The cells were injected at the sides of the wells, opposite the previously made holes. The efficiency of the injection and the cell spreading were controlled using a light microscope. Then, excess medium was removed from the punched holes using a pipette.

Prepared slides were placed on a pre-heated microscope stage (Olympus IX83 inverted widefield microscope, U-HGLGPS light source, and QImaging Retiga-2000R mono camera) equipped with a live imaging chamber (Okolab) set for standard culture conditions (37°C, 5% CO_2_). The imaging was initiated 30 min after the injection of the cells. The cells under the agarose were imaged using 10x magnification (CPLFLN PH objective, Olympus) in phase contrast and the blue fluorescence channel in parallel (1 frame per 10 s) for a total of 60 frames. The same lamp intensity, exposition, and camera gain were used for all treatments in a replicate.

### Tracking

Acquired time-lapse image series saved as *.vsi* files were first pre-processed in FIJI software (version 2.3.0/1.53f51) (Schindelin et al., 2012) using a custom macro (Supplementary Table 2). The macro uses the Image Stabilizer plugin (K. Li, “The image stabilizer plugin for ImageJ,” http://www.cs.cmu.edu/∼kangli/code/Image_Stabilizer.html, February 2008) to normalize shaking of the microscope stage. After the image data pre-processing, the cells were tracked based on the blue fluorescence channel using the TrackMate plugin (v7.5.1)^33^. The tracking using the LoG detector and simple LAP tracker was set up as follows: estimated object diameter: 10 μm, quality threshold: 0.2, linking max distance: 10 μm, gap-closing max distance: 15 μm, and gap-closing max frame gap: 2. For the MINO cell line, the estimated object diameter had to be increased to 12 µm to ensure reliable spot detection due to the larger nuclei compared to other tested cell lines. For primary CLL cells, the estimated object diameter was set to 9 μm and the quality threshold to 0.1 (except for patient #6, where due to a low signal, the threshold had to be set to 0.52). Only complete trajectories (containing all 60 spots/timepoints) were chosen for further analysis. The migration parameters were used as calculated in the TrackMate “*Tracks*” results table (Tinevez et al., 2017). For the calculation of confinement ratio decay and mean square displacement (MSD) from spot coordinates (“*Spots*” TrackMate results table), we used the DIPER MS Excel macro (Gorelik and Gautreau, 2014).

### Transwell assay

We have used 5-µm pore polycarbonate transwell inserts for 24-well plates (Corning, cat. no. CLS3421). The inserts were coated with 2 μg/ml recombinant VCAM1 in 0.1% BSA/PBS or just with BSA/PBS as a negative control overnight at 4°C. On the next day, before the loading of the cells, the coating solution was completely removed. Then, 600 µl of complete RPMI medium with supplements (200 ng/ml recombinant CCL19, 10 µM PF670462, or equal volumes of 0.1% BSA/PBS or DMSO) as indicated in the description of Figure 4 were first pipetted to an empty 24-well plate, and then the empty transwell inserts were gently placed into the wells. After that, 50 µl of cell suspension in the complete RPMI medium were pipetted onto the inserts at a density of 300,000 cells/well. Finally, another 50 µl of complete RPMI medium were added to the insert, which also contained 20 µM PF670462 or an equal volume of DMSO dependent on the experiment condition to achieve an equal inhibitor concentration in the upper well of the insert. The transwell plates were then incubated for 3 h at standard culture conditions (37°C, 5% CO_2_). After that, the inserts were removed, and the transmigrated cells in the lower well counted using the flow cytometer. The amounts of cells were then normalized to the “input” condition, which contained the same volume of cells; these were, however, pipetted directly to the lower well of the transwell system and thus represented an ideal state in which all loaded cells transmigrated. The migration index (MI) was calculated as the number of transmigrated cells in individual conditions divided by the number in the unstimulated condition. Thus, the MI value for the unstimulated condition equals 1, and other values represent the fold change in migration.

### Migration data visualization and statistics

For visualization and statistical comparison of cell migration parameters, we used Violin SuperPlots (Lord et al., 2020; Kenny and Schoen, 2021). According to recommendations summarized by Lord et al. (2020), we used only the median values from each replicate for statistical testing, instead of pooling whole datasets, in order to avoid the artificial increase of the sample number and the batch effect. The medians of the measurements are represented as circles inside the strips of the SuperPlots, representing the distribution of individual replicates. The black error bar represents the global mean and SD. For the visualization of confinement ratio decay and MSD, we followed the visualization generated by the DIPER MS Excel macro, and the values shown in the graph are further described in the Figure 2 legend. For visualization of the transwell assay data, we used GraphPad Prism 8 software. The box plots show minimum/maximum with individual replicate values. We used one-way ANOVA and (where applicable) the Tukey post hoc test for statistical testing of the data.

### Detailed live imaging and visualization of the polarized endocytic processes

For the detailed time-lapse imaging shown in Figure 1 and Supplementary Movies S1–S14, we used the same microscope as for under-agarose migration analysis (see above) and used 40x magnification (LUCPLFLN PH objective, Olympus) in the bright-field mode. The cells were imaged at an interval of 2 s for a total of 80 frames either confined under agarose (see above) or unconfined (in the RPMI medium). In both setups, we used dishes with the same surface treatment (µ-Slide 8 Well ibiTreat, ibidi).

For the visualization of the cell membrane and endocytic vesicles, we used CellBrite Steady 650 stain (Biotium, cat. no. 30108). The cells were harvested the same way as for the regular under-agarose migration experiment (see above); only this time, instead of staining the nuclei, we resuspended the cells in 400 µl of complete RPMI medium supplemented with 0.4 µl of the stain and incubated them at standard culture conditions (37°C, 5°C) for 30 min. After that, the cells were centrifuged and washed with 1 ml of PBS and then resuspended in a stain-free complete RPMI medium. Then, the cells were injected under agarose as described earlier. Upon the injection, the slide with agarose was placed in the preheated on-stage incubator (UNO-T-H-CO2, Okolab) and further incubated for 30 min at standard culture conditions. For this experiment, we used the Leica DMI6000 B inverted microscope in the widefield mode equipped with the EL6000 light source (Leica) and the ORCA-Flash4.0 V3 Digital CMOS camera C13440-20CU (Hamamatsu). We used 63x magnification (HC PL FLUOTAR L 63x/0.70 DRY objective, Leica Microsystems), and the cells were imaged at 10 s interval for a total of 40 frames. For quantification of the vesicle dispersion, we used the same microscope, only this time at 40x magnification (HC PL FLUOTAR L 40x/0.60 DRY objective, Leica Microsystems), to capture more cells in the FOV and be able to easily focus on whole cells. At least four randomly selected FOVs were captured for each replicate and experimental condition in the red fluorescence channel and brightfield mode (for visual reference). Due to the uneven background, we had to cut the acquired images of FOVs into smaller parts, containing one cell at a time. The smaller images were then duplicated, and one copy was segmented according to the red fluorescence channel using a FIJI macro (Supplementary Table 2). The segmentation process included preprocessing by enhancing contrast and applying a Gaussian blur (sigma = 2), and then the Huang thresholding method (Huang and Wang, 1995) was used to get a binary image of the shape of the cell. The cells that were in contact with other cells or could not be segmented due to an uneven background were excluded from analysis. For further quantification, we used two different approaches.

In the first, the resulting ROI was applied to the second copy of the image and converted to an array of smaller square ROIs using a script published at the image.sc forum (Rueden et al., 2019) by Christian Evenhuis (https://forum.image.sc/t/subdividing-cell-perimeter-into-multiple-roi/28142, September 2020). Based on the size of the vesicles and the used magnification, we used a square size of 3 ×3 pixels (0.238 µm^2^). We then measured the median signal intensity in these squares. The resulting table was then processed using an R-script (Supplementary Table 3); using it, we first filtered the squares that contained the vesicle signal based on intensity. As the cells differed in intensity, we set the threshold as a 2x minimum intensity value in every individual cell. We then computed all mutual distances between any two centroids of positive squares in the given cell. We then calculated the median value from the distances for every individual cell. In the second approach, we used the FIJI function “*Find maxima…*” (prominence value > 200) to identify local maxima inside the cell, the area of which was defined by the previously obtained ROI. We then exported the coordinates of the identified maxima and calculated their mutual distances in the same way as described in the first approach. We then also used the median value of all distances for individual cells.

### Cell transfection

MEC1 cells were taken from the culture, centrifuged and washed with PBS two times, and then resuspended in 100 µl of PBS. The cell suspension was mixed with 10 µl of 2 µg/µl plasmid and electroporated using the Neon transfection system (Invitrogen), using the pulse setting 1,200/20/2 (pulse voltage/pulse width/number of pulses). After electroporation, the cells were cultivated overnight in RPMI supplemented with 20% FBS. For the visualization of endocytic machinery, we used plasmid GFP-rab11 WT (Addgene, cat. no. 12674) (Choudhury et al., 2002).

### Preparation of CK1ε-deficient MEC-1 cells by CRISPR/Cas9

For transfection (see above), we used the plasmid backbone of pSpCas9(BB)-2A-GFP (PX458) (Addgene #48138) (Ran et al., 2013) containing the cloned sequence to be transcribed to gRNA (AAG​TTC​TAC​AAG​ATG​ATG​CA), targeting CK1ε exon #3. The day following the transfection, the cells were taken from the culture, centrifuged and washed, and then resuspended in PBS with 1% FBS. The GFP-positive cells were then sorted using FACS into round-bottom 96-well plates (1 cell/well) containing 70 µl of RPMI media with 20% FBS in every well. The plates were then kept in the incubator for 2–3 weeks until the clones multiplied enough to be transferred to a larger volume and tested. The gene knockout in the arising clones was then verified by sequencing as described previously (Pavlova et al., 2019). Loss of protein expression was verified by western blot.

### Flow cytometry analysis of the surface expression of integrins and chemokine receptors

The cells (500,000) were cultivated in a fresh medium overnight. For the testing of the inhibitors shown in Supplementary Figure S6, DMSO or the indicated inhibitors (final concentration 10 µM) were added to the culture 1 h before the samples were harvested. The samples were washed with PBS and resuspended in staining solution (2% FBS in PBS), containing a mixture of antibodies designated for flow cytometry (see Supplementary Table 1). After 15 min of incubation at RT, samples were washed in PBS and processed by a spectral cytometer Cytek Northern Lights 300 (405, 488, and 647 nm lasers, 16V, 14B, and 8-R channels, Cytek Biosciences, CA, United States). The AbC Total Antibody Compensation Bead Kit (Fisher Scientific) was used for the preparation of reference controls for unmixing. For the unmixing of each cell line, the respective unstained control was used in the SpectroFlo software (Cytek). Data were analyzed using FlowJo v10.8.1 software (BD). Dead cells and doublets were excluded from the analysis.

FlowSOM based on the surface expression of chemokine receptors, integrins, and ROR1 was used for the analysis of the intrinsic diversity of cell lines on separate replicates. Analysis was carried out using an R script published by Quintelier et al. (2021). Unmixed *.fcs* files without debris and doublets were loaded. Data were transformed (logicle transformation) and normalized using the CytoNorm algorithm (Van Gassen et al., 2020). The model was trained and verified using manual gating in FlowJo. Metaclusters and clusters were created using the minimal spanning tree method. Clusters were visualized and mapped on tSNE using the same protocol. HG-3 and MINO cells were assigned to more than one cluster due to their heterogeneity. They were subjected to separate clustering. Expression of panel of surface markers was visualized as a heatmap.

### Fluorescence-activated cell sorting

HG-3 cells were stained using the ROR1-PE antibody (cat. no. 130-098-317, Miltenyi Biotec) using the same staining protocol as described earlier. The PE-positive cells were then sorted using the FACSAria III sorter (BD) from the PE-negative, resulting in two subpopulations that were then cultivated separately. ROR1 expression has been routinely tested after sorting using flow cytometry.

### Western blot analysis

For the BCR stimulation experiment, 3 million cells from every tested cell line were taken from the culture, washed with PBS, and resuspended in 300 µl of fresh PBS. Then, the cell suspension was divided into three Eppendorf tubes (100 µl/tube) and placed on a pre-warmed block heater and incubated for 5 min at 37 °C. Then, the cells are stimulated directly in the heater by the addition of 100 µl of cold treatment solution. In the control condition, the cells were treated with only PBS, in the second with H_2_O_2_ (final concentration 3.3 mM) diluted in PBS, and in the third with both the same concentration of H_2_O_2_ and anti-human IgM (Southern Biotech, cat. 2022-01, final concentration 10 μg/ml). The treatment solutions were always mixed fresh directly before the experiment and kept on ice. The cells were incubated with the treatment for 4 min and then centrifuged (400 g, 5 min, 4°C). Then, the supernatant was removed, and the cell pellets were lysed in 100 µl of cold 1% SDS lysis buffer (1% SDS, 10% glycerol, 60 mM Tris pH 6.8) supplemented with phosphatase and protease inhibitor cocktails (Merck). For the chemokine stimulation experiment, 2.5 million cells from each of the tested cell lines were taken from the culture, washed with PBS, and resuspended in 1 ml of fresh RPMI. The cells were then incubated for 1 h in the incubator and then treated with the chemokine (or an equal volume of 0.1% BSA in PBS in the control condition) at a final concentration of 200 ng/ml. The cells were incubated with the treatment for another 1 h and then harvested, centrifuged, washed with PBS, and lysed using 1% SDS lysis buffer as described earlier.

For the CRISPR-Cas9 knockout clone validation, 2.5 million cells were taken from the culture of wild-type MEC1 cells and each of the tested clones. The cells were centrifuged, washed with PBS, and then lysed as described earlier.

The cell lysates were then sonicated for 2 min, and the protein concentration of samples was measured using the DC Protein Assay kit (Bio-rad) and normalized. β-Merkaptoethanol (final concentration: 5%) and bromophenol blue (final concentration: 0.004%) were added to each sample. After that, the samples were heated to 95°C for 5 min. After that, the SDS-PAGE was performed using the Mini-PROTEAN Tetra Cell vertical electrophoresis system (Bio-rad). After SDS-PAGE, the samples were transferred by the wet transfer method to activated Immobilon-P PVDF Membranes (Sigma Aldrich). The membranes were then blocked using 5% nonfat milk in wash buffer (100 mM NaCl, 0.08% Tween 20, and 10 mM Tris pH 7.6) for 1 h at room temperature with shaking. In the experiments with BCR stimulation, we used 3% BSA (Serva) instead of nonfat milk for the whole procedure. After the blocking, the membranes were cut into strips according to the PageRuler protein ladder (Thermofisher) and incubated with primary antibodies (dilution 1:500 in 5% nonfat milk/3% BSA) overnight at 4°C. Primary antibodies used in this article were as follows: mouse anti-CK1ε (BD Transduction Laboratories, cat. no. 610445), rabbit anti-alpha-tubulin (Cell Signaling Technology, cat. no. cs-5335S), rabbit anti-phospho-Syk Tyr525/526 (Cell Signaling Technology, cat. no. 2711), and rabbit anti-β-actin (Cell Signaling Technology, cat. no. 4970). After this step, the membranes were washed three times with wash buffer (15 min each step) and then incubated for 1 h with secondary antibodies at room temperature. We used Anti-Rabbit IgG (whole molecule)–Peroxidase antibodies and Anti-Mouse IgG (whole molecule)–Peroxidase antibodies produced in goat (Sigma-Aldrich, cat. no. A0545 and A4416). After the incubation, the membranes were washed again three times, and then the antibody signal was detected by the addition of Immobilon Western Chemiluminescent HRP Substrate (Sigma-Aldrich, cat. no. WBKLS0100) in the FUSION SL chamber (Vilber Lourmat).

## Data Availability

The raw data supporting the conclusion of this article will be made available by the authors, without undue reservation. All data files related to image analysis experiments will be uploaded to The Dryad Digital Repository (https://datadryad.org/) upon publishing. The flow cytometry data will be uploaded to FlowRepository database (https://flowrepository.org/) upon publishing.

## Supplementary material

SUPPLEMENTARY MOVIE S1 Representative video of HG-3 cells’ migration in the under-agarose assay, imaged at the interval of 2 s for 80 frames.

SUPPLEMENTARY MOVIE S2 Representative video of MEC-1 cells’ migration in the under-agarose assay, imaged at the interval of 2 s for 80 frames.

SUPPLEMENTARY MOVIE S3 Representative video of MAVER-1 cells’ migration in the under-agarose assay, imaged at the interval of 2 s for 80 frames.

SUPPLEMENTARY MOVIE S4 Representative video of MINO cells’ migration in the under-agarose assay, imaged at the interval of 2 s for 80 frames.

SUPPLEMENTARY MOVIE S5 Illustrative video of the cell tracking used in this study. The cells are imaged in two channels: brightfield—for visual check of the cell behavior; blue channel—for the tracking itself. As the nuclei represent the majority of the cell volume in lymphoid cells and mostly express a relatively circular shape, we decided to use them as a representation of the cell center for cell tracking. The centroids of the nuclei stained with Hoechst 33342 are tracked by the TrackMate plugin for FIJI.

SUPPLEMENTARY MOVIE S6 Representative video of the oscillating behavior of MINO cells shown with the resulting tracks. Cells were imaged at 10 s interval for a total of 60 frames.

SUPPLEMENTARY MOVIE S7 Representative video of the migratory behavior of the HG-3 cells in the control condition (treated by DMSO). Imaged at an interval of 10 s for 120 frames. Blue: Hoechst 33342.

SUPPLEMENTARY MOVIE S8 Representative video of the migratory behavior of the HG-3 cells treated with 10 μM PF670462 for 30 minutes. Imaged at an interval of 10 s for 120 frames. Blue: Hoechst 33342.

SUPPLEMENTARY MOVIE S9 Representative video of the migratory behavior of the HG-3 cells treated by 10 μM Y27632 for 30 minutes. Imaged at an interval of 10 s for 120 frames. Blue: Hoechst 33342.

SUPPLEMENTARY MOVIE S10 Representative video of the migratory behavior of the MEC-1 cells in the control condition (treated by DMSO). Imaged at an interval of 10 s for 120 frames. Blue: Hoechst 33342.

SUPPLEMENTARY MOVIE S11 Representative video of the migratory behavior of the MEC-1 cells treated with 10 μM PF670462. Imaged at an interval of 10 s for 120 frames. Blue: Hoechst 33342.

SUPPLEMENTARY MOVIE S12 Representative video of the migratory behavior of the MEC-1 cells treated by 10 μM Y27632 for 30 minutes. Imaged at an interval of 10 s for 120 frames. Blue: Hoechst 33342.

SUPPLEMENTARY MOVIE S13 Time-lapse videos of endocytic polarity experiments. 1) MEC-1 cell phenotypes in the control (treated by DMSO) condition. 2) Same cell line treated with 10 μM PF670462. 3). Same cell line treated by 10 μM Y27632. Black signal: Cellbrite Steady 650 plasma membrane stain. Imaged at 10 s interval for 40 frames each.

SUPPLEMENTARY MOVIE S14 Representative video of the migratory behavior of the MAVER-1 cells in the unstimulated condition. Imaged at an interval of 10 s for 60 frames.

SUPPLEMENTARY MOVIE S15 Representative video of the migratory behavior of the MAVER-1 cells stimulated by the combination of a VCAM-coated surface and 200 ng/ml CCL19. Imaged at an interval of 10 s for 60 frames.

SUPPLEMENTARY FIGURE S1 Responsiveness of the tested cell lines to BCR stimulation as detected by the increase in the phosphorylated form of kinase Syk. Actin signal shown as a loading control. Representative images from three biological replicates.

SUPPLEMENTARY FIGURE S2 Total distance traveled by HG-3 cells upon CK1 or ROCK inhibition. **(A)** Statistics: one-way ANOVA, *n* = 4, *p* = 0.002, and the post hoc Tukey test. **(B)** Total distance traveled by MEC1 cells in the same experimental setup. Statistics: one-way ANOVA, *n* = 4, *p* < 0.001, and the post hoc Tukey test. The data shown in **(A–B)** are derived from the experiments shown in Figures 3A and 3B, respectively. **(C)** Effect of combined treatment with CK1 and ROCK inhibitors on the HG-3 cell line on max. distance traveled parameter. Statistics: one-way ANOVA, *n* = 3, *p* < 0.001, and post hoc Tukey test. **(D)** Confinement ratio of HG-3 cell lines from the same experiment. Statistics: one-way ANOVA, *n* = 3, *p* < 0.001, and the post hoc Tukey test. **(E)** Max. distance traveled by MEC-1 cells in the same experimental setup. Statistics: one-way ANOVA, *n* = 3, *p* < 0.001, and the post hoc Tukey test. **(F)** Confinement ratio of MEC1 cells from the same experiment. Statistics: one-way ANOVA, *n* = 3, *p* < 0.001, and the post hoc Tukey test. Numbers of cells (tracks) quantified in **(C–D)**, left to right: CTR (55, 113, 75), PF670462 (63, 207, 128), Y27632 (71, 74, 93), and combination (69, 152, 45). **(E–F)**, left to right: CTR (112, 182, 198), PF670462 (38, 63, 99), Y27632 (36, 84, 85), and combination (185, 173, 154). SuperPlot settings in all panels are consistent with those described in Figure 2A.

SUPPLEMENTARY FIGURE S3 Effects of CK1 inhibitors PF670462 and MU1742 and a ROCK inhibitor Y27632 on the basal migratory properties of the tested MCL cell lines. **(A)** Maximum distance traveled by MAVER-1 cells treated with 10 μM of indicated inhibitors or equal volume of DMSO (CTR). Statistics: one-way ANOVA, *n* = 4, and *p* = 0.05. **(B)** Total distance traveled by MAVER-1 cells in the same experiment. Statistics: one-way ANOVA, *n* = 4, and *p* = 0.121. **(C)** Analysis of MAVER-1 migratory persistence under the effects of CK1 and ROCK inhibitors. Left: decay of CR over the period of tracking. Center: CR values at the last frame of the tracking period (t = 590 s). Statistics (one-way ANOVA, *n* = 4, *p* = 0.378). Right: MSD analysis. **(D)** Effects of CK1 and ROCK inhibition on basal migratory properties of the MINO cell line as measured by the max. distance traveled parameter. Statistics: one-way ANOVA, *n* = 4, and *p* = 0.060. **(E)** Total distance traveled of MINO cells from the same experiment. Statistics: one-way ANOVA, *n* = 4, *p* = 0.008, and the post hoc Tukey test. **(F)** Analysis of MINO migratory persistence under the effect of CK1 and ROCK inhibitors. Left: decay of CR over the period of tracking. Center: CR values at the last frame of the tracking period (t = 590 s). Statistics: one-way ANOVA, *n* = 4, *p* = 0.0017, and the post hoc Tukey test. Right: MSD analysis. SuperPlot settings in all panels are consistent with those described in Figure 2B. Number of cells (tracks) measured in A–C (same experiment): CTR (66, 70, 271, 53), PF670462 (113, 38, 25, 57), MU1742 (76, 49, 220, 89), and Y27632 (89, 65, 108, 61). D–F (same experiment): CTR (123, 48, 55, 49), PF670462 (48, 61, 135, 129), MU1742 (127, 48, 213, 272), and Y27632 (128, 114, 195, 191).

SUPPLEMENTARY FIGURE S4 CellBrite vesicle distribution quantification. **(A)** Two approaches tested for the quantification of vesicle distribution in MEC-1 cells. 1. Image data are pre-processed and thresholded to obtain binary masks of the cell outline. This step is common for both approaches. White: CellBrite 650 Steady fluorescence signal. In first approach (2a), the ROI representing the shape of the cell (yellow) is subdivided into smaller squares of equal size (for this analysis, we used 3x3 pixels). Then, (3a) the array of ROIs is applied to the original image and the median signal intensity is measured in every square. The acquired data are filtered based on the relative intensity, the squares with values at least two times higher than the minimum measured value are considered as positive (red). Mutual distance between centroids of all identified positive squares in one cell is calculated (4a). In the second approach (2b), the ROI obtained from thresholding is applied to the original image, and local maxima (yellow dots/stars) are identified using the FIJI command “Find Maxima…” Next (3b), the mutual distance between all identified points in one cell is calculated from their coordinates. **(B)** Results of quantification obtained by the (first) approach using subdivision of ROIs presented as median distance between positive squares. Statistics: one-way ANOVA, n = 4, p < 0.001, and the post hoc Tukey test. **(C)** Results of quantification obtained by the (second) approach using local maxima, presented as median distance between identified maxima. Statistics: one-way ANOVA, *n* = 4, *p* < 0.001, and the post hoc Tukey test. SuperPlot settings in both panels are consistent with those described in Figure 2B. Number of analyzed cells in B–C (derived from the same image data): CTR (87, 74, 38, 67), PF670462 (92, 96, 67, 79), and Y27632 (58, 72, 62, 56).

SUPPLEMENTARY FIGURE S5 Effect of positive stimulation by CXCL12 (SDF1a) on the migration of leukemic cell lines. The cells were stimulated by 200 ng/ml recombinant CXCL12 in combination with VCAM1 coating or 10 μM PF670462. For the treatment setup, please see the scheme in the center of the figure. **(A–B)** Response of the HG-3 cell line to CXCL12, VCAM1 coating, and CK1 inhibition as quantified by maximum distance traveled (A) (statistics: one-way ANOVA, n = 4, p < 0.001, and the post hoc Tukey test) and confinement ratio **(B)** (statistics: one-way ANOVA, *n* = 4, *p* = 0.001, and the post hoc Tukey test). **(C–D)** Response of MEC-1 cells in the same experimental setup as quantified by maximum distance traveled **(C)** (statistics: one-way ANOVA, n = 5, p < 0.001, and the post hoc Tukey test) and confinement ratio **(D)** (statistics: one-way ANOVA, *n* = 5, *p* = 0.003, and the post hoc Tukey test). **(E–F)** Response of MAVER-1 cells to chemokine stimulation in the same experimental setup. (E) Maximum distance traveled, statistics: one-way ANOVA, *n* = 4, *p* = 0.001, and the post hoc Tukey test. **(F)** Confinement ratio, statistics: one-way ANOVA, *n* = 4, *p* = 0.001, and the post hoc Tukey test. (G-H) Response of MINO cells in the same experimental setup. **(G)** Maximum distance traveled, statistics: one-way ANOVA, *n* = 4, *p* = 0.006, and the post hoc Tukey test. **(H)** Confinement ratio, statistics: one-way ANOVA, n = 4, p < 0.001, and the post hoc Tukey test. SuperPlot settings in all panels are consistent those described in Fig. 2B. Numbers of cells (tracks) measured in **(A–B)** (same experiment), left to right: 107, 64, 250, 149; 98, 115, 151, 81; 138, 64, 72, 161; 110, 88, 111, 133; 91, 188, 137, and 122. **(C–D)** (same experiment), left to right: 111, 99, 105, 133, 113; 229, 85, 134, 121, 107; 286, 87, 158, 150, 77; 279, 219, 166, 97, 136; 195, 128, 250, 161, and 149. **(E–F)** (same experiment), left to right: 109, 130, 136, 117; 161, 154, 203, 93; 231, 103, 125, 57; 198, 129, 222, 127; 115, 180, 159, and 133. **(G–H)** (same experiment), left to right: 183, 102, 177, 105; 170, 161, 158, 236; 214, 176, 208, 148; 134, 139, 90, 192; 183, 195, 138, and 220. **(I)** Western blot screening of CK1ε; expression after 1 h stimulation with 200 ng/ml recombinant chemokines CXCL12 and CCL19 in tested cell lines. α-Tubulin signal shown as a loading control. Representative image from three biological replicates.

SUPPLEMENTARY FIGURE S6 Effect of positive stimulation by human recombinant chemokines CCL19 or CXCL12 (200 ng/ml) and VCAM1 coating on the migration properties of primary CLL cells from six independent patient samples. SuperPlots show individual cells in samples as spots, and darker spots show the median value. Migration properties are demonstrated using the total distance traveled, **(A)** confinement ratio **(B)**, and maximum distance traveled **(C)** parameters. Numbers of cells (tracks) analyzed in **(A–C)** (derived from the same experiment), left to right: patient #1 (72, 64, 60, 57, 36, and 60), patient #2 (287, 293, 102, 79, 186, and 264), patient #3 (320, 626, 193, 300, 392, and 188), patient #4 (118, 63, 90, 44, 74, and 67), patient #5 (504, 497, 262, 129, 208, 256), and patient #6 (56, 123, 113, 59, 44, and 76).

SUPPLEMENTARY FIGURE S7 Statistical analysis of data from the panel of measured markers shown in Fig. 5A–B. Medians of the median fluorescence intensities (MFI) for individual markers were compared among the tested cell lines. Individual dots represent biological replicates; error bars: mean and SD. **(A)** CCR7, statistics: one-way ANOVA, *n* = 3, and *p* = 0.0981. **(B)** CD29, statistics: one-way ANOVA, *n* = 3, *p* = 0.0073, and the post hoc Tukey test. **(C)** CD49d, statistics: one-way ANOVA, *n* = 3, *p* = 0.0084, and the post hoc Tukey test. **(D)** CXCR4, statistics: one-way ANOVA, *n* =3, *p* = 0.0012, and the post hoc Tukey test. **(E)** CD11a, statistics: *n* =3 and *p* = 0.4550. Negative values arise from very low signal values and subsequent signal compensation. **(F)** CD18, statistics: *n* = 3, *p* = 0.0003, and the post hoc Tukey test. **(G)** ROR1, statistics: *n* = 3, *p* = 0.0006, and the post hoc Tukey test.

SUPPLEMENTARY FIGURE S8 Screening of possible effects of the tested CK1 and ROCK inhibitors on the level of surface expression of chemokine receptors, integrins, and ROR1 in tested cell lines. The cells were incubated for 1 h with 10 µM of the indicated inhibitor or an equal volume of DMSO (CTR). Individual dots represent medians of median fluorescence intensity values, which were used for statistical analysis. Error bars: mean and SD. Statistical testing by one-way ANOVA was performed for individual cell lines. **(A)** CCR7. Statistics: *n* = 3; *p* values: HG-3 (0.1793), MEC-1 (0.968), MAVER-1 (0.9345), and MINO (0.8969). **(B)** CD29. Statistics: *n* = 3; *p* values: HG-3 (0.5667), MEC-1 (0.9693), MAVER-1 (0.9890), and MINO (0.4434). **(C)** CD49d. Statistics: *n* = 3; *p* values: HG-3 (0.1257), MEC-1 (0.9780), MAVER-1 (0.9793), and MINO (0.4563). **(D)** CXCR4. Statistics: *n* = 3; *p* values: HG-3 (0.7831), MEC-1 (0.9873), MAVER-1 (0.4853), and MINO (0.9837). **(E)** CD11a. Statistics: *n* = 3; *p* values: HG-3 (0.9050), MEC-1 (0.9981), MAVER-1 (0.9898), and MINO (0.9879). **(F)** CD18. Statistics: *n* = 3; *p* values: HG-3 (0.4592), MEC-1 (0.3708), MAVER1 (0.9874), and MINO (0.9396). **(G)** ROR1. Statistics: *n* = 3; *p* values: HG-3 (0.9816), MEC-1 (0.9998), MAVER-1 (0.9687), and MINO (0.9475).

SUPPLEMENTARY FIGURE S9 Migration analysis of subpopulations of HG-3 cell line based on their ROR1 expression. **(A)** Flow cytometry validation of the ROR1 expression profiles of sorted HG-3 subpopulations. Up: unsorted culture containing both subpopulations; middle: ROR1^+^ sorted subpopulation, bottom: ROR1^-^ sorted subpopulation. **(B)** Comparison of total distance traveled by HG-3 ROR1 subpopulations and unsorted culture. Statistics: one-way ANOVA, *n* = 3, *p* = 0.042, and the post hoc Tukey test. **(C)** Maximum distance traveled. Statistics: one-way ANOVA, *n* = 3, p = 0.035, and the post hoc Tukey test. **(D)** Confinement ratio. Statistics: one-way ANOVA, *n* = 3, *p* = 0.039, and the post hoc Tukey test. SuperPlot settings are consistent with those used in Fig. 2A. Numbers of cells (tracks) measured in **(B–D)** (data derived from the same experiment): unsorted (204, 136, 80), ROR1^-^ (94, 96, 176), and ROR1^+^ (124, 106, 274).

SUPPLEMENTARY TABLE S1 Table of antibodies used for flow cytometry analysis.

SUPPLEMENTARY TABLE S2 FIJI macros used for pre-processing of the time-lapse data for tracking analysis and for segmentation of cellular outlines from CellBrite data.

SUPPLEMENTARY TABLE S3 R scripts for the calculation of the mutual distances between identified vesicles (both approaches).
