# Supplementary figures and images for: Role of casein kinase 1 in the amoeboid migration of B-cell leukemic and lymphoma cells: A quantitative live imaging in the confined environment
Source: Front Cell Dev Biol. 2022 Dec 6;10:911966. doi: 10.3389/fcell.2022.911966 (PMC9763939; doi:10.3389/fcell.2022.911966)

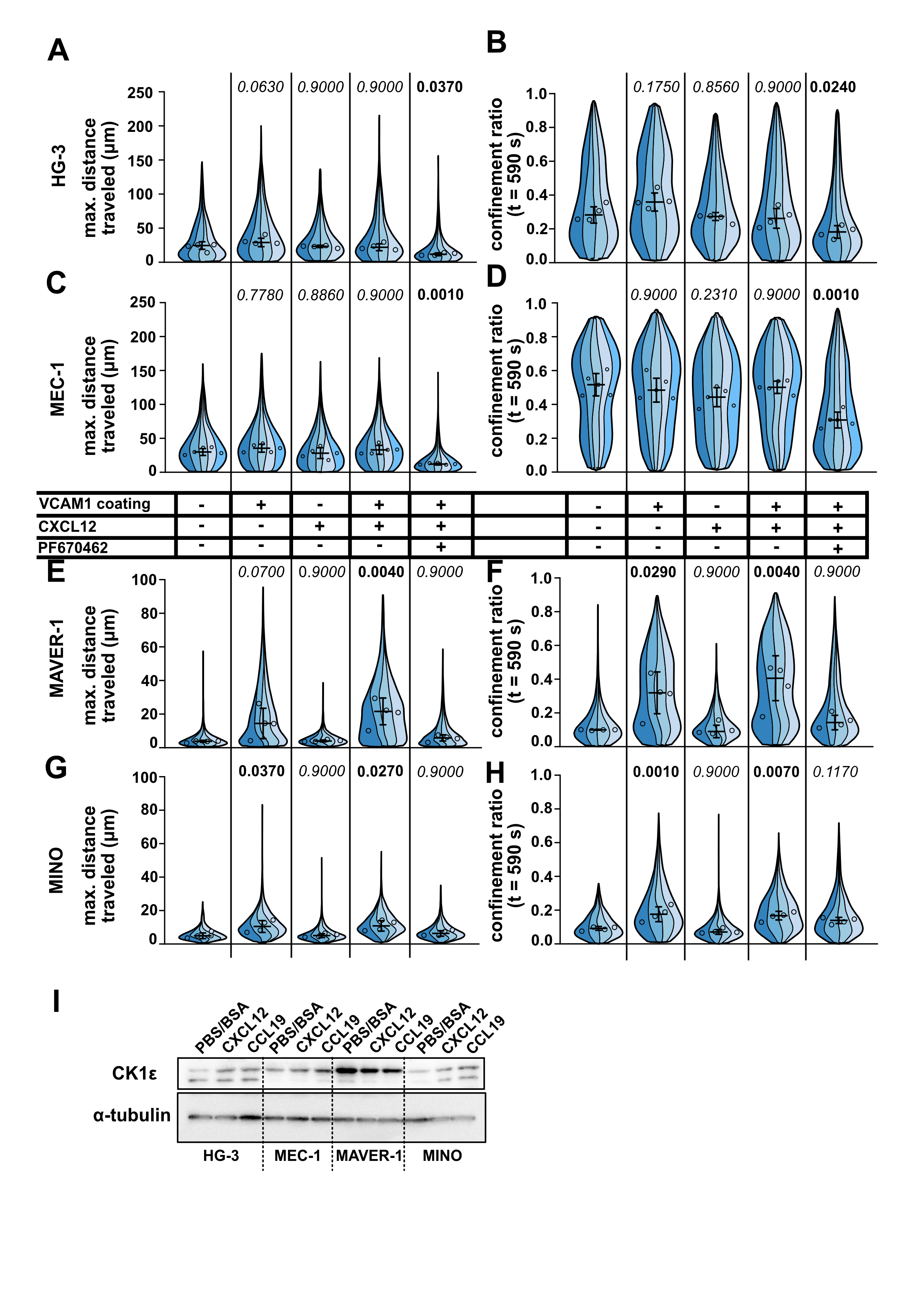

Supplement: Supplementary file 2 [file Image5.jpg]

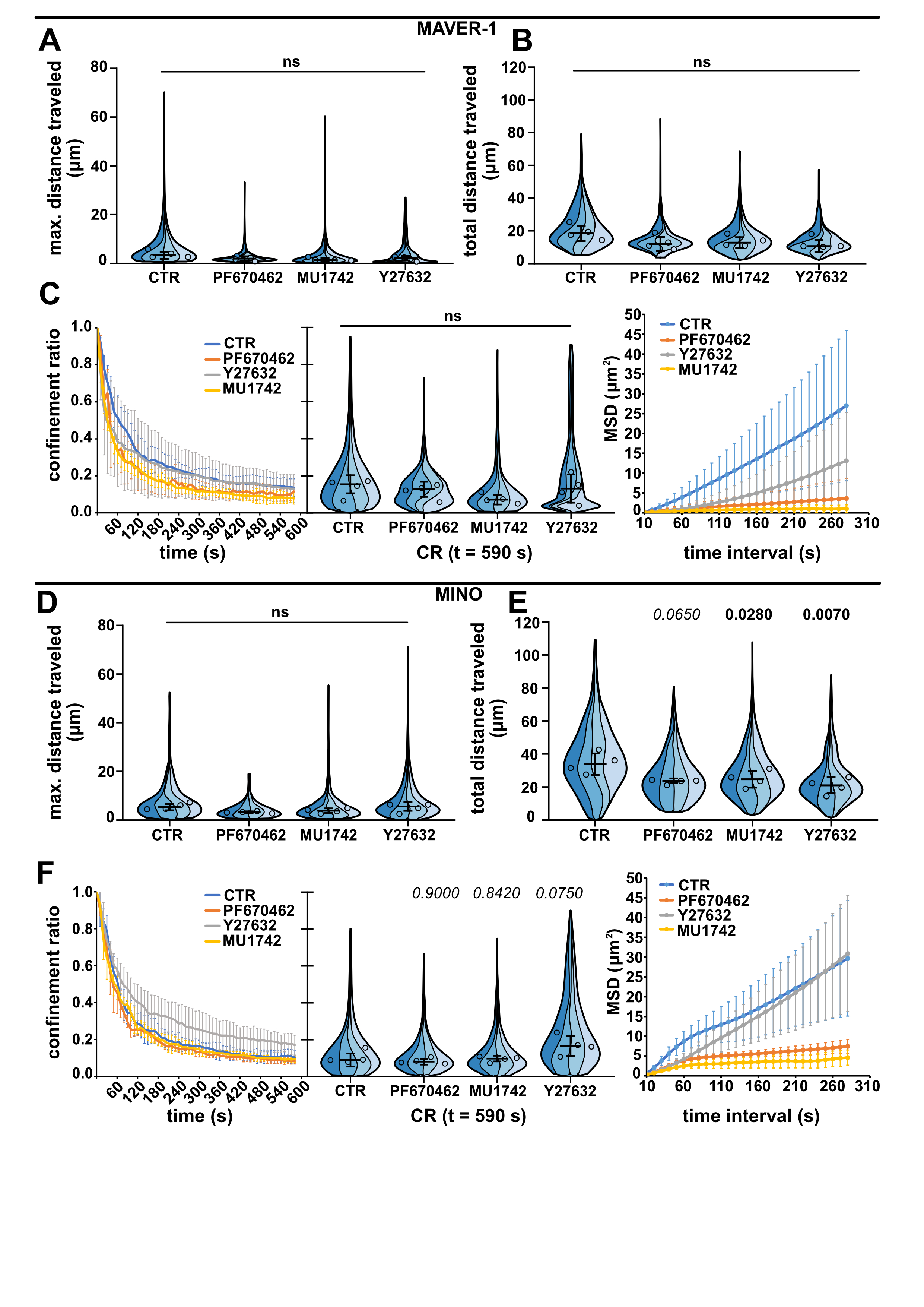

Supplement: Supplementary file 3 [file Image3.JPEG]

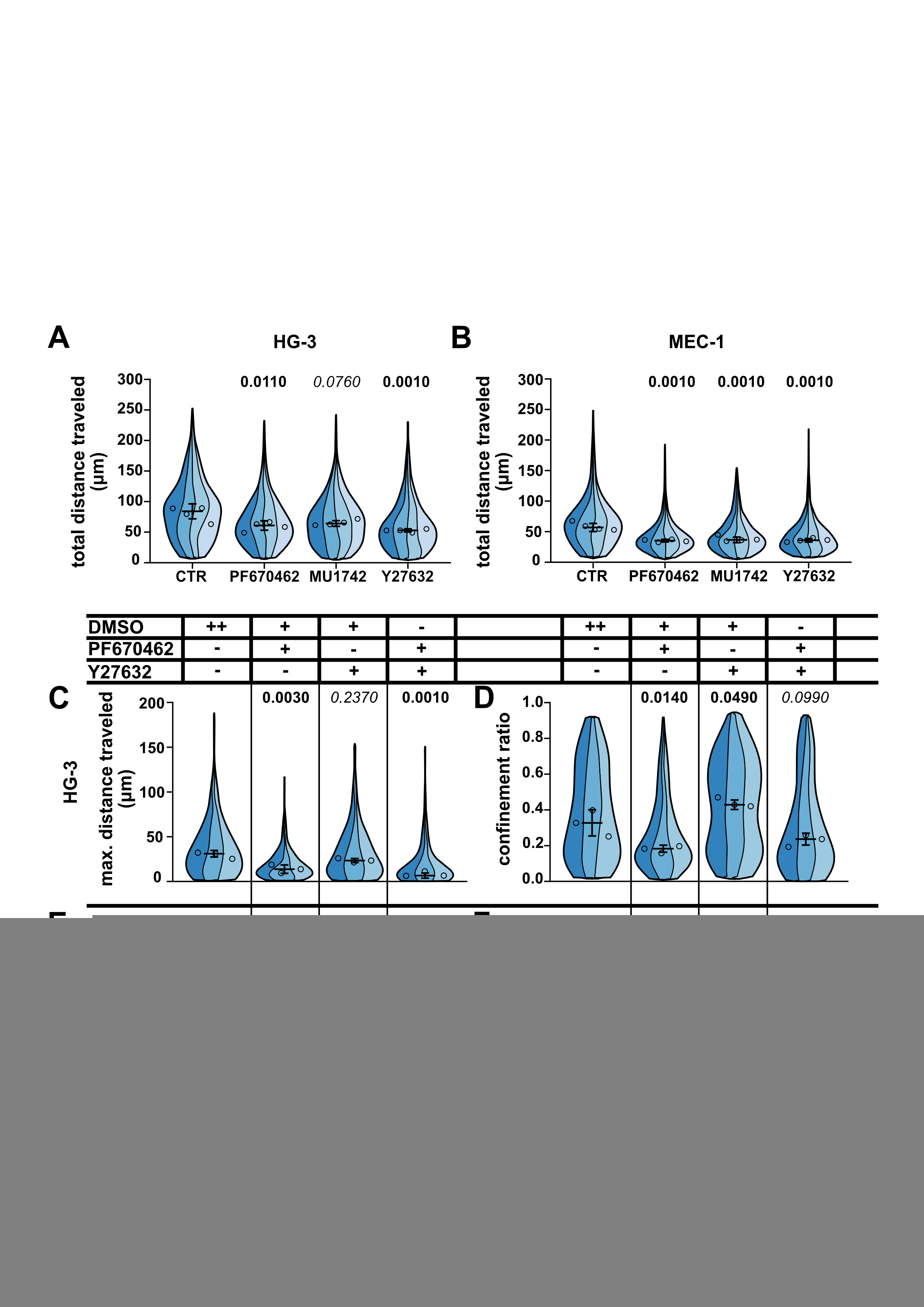

Supplement: Supplementary file 8 [file Image2.jpg]

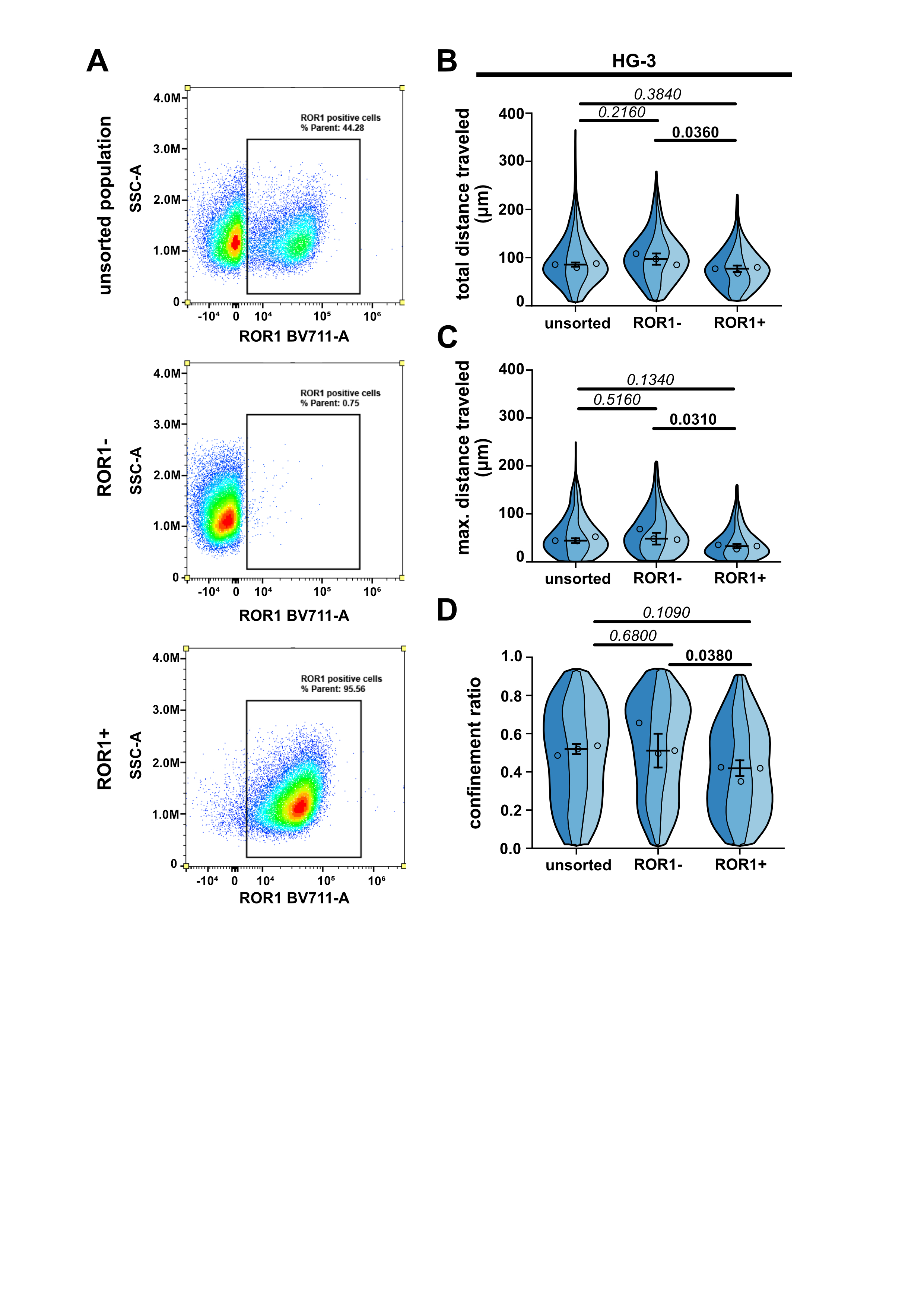

Supplement: Supplementary file 11 [file Image9.JPEG]

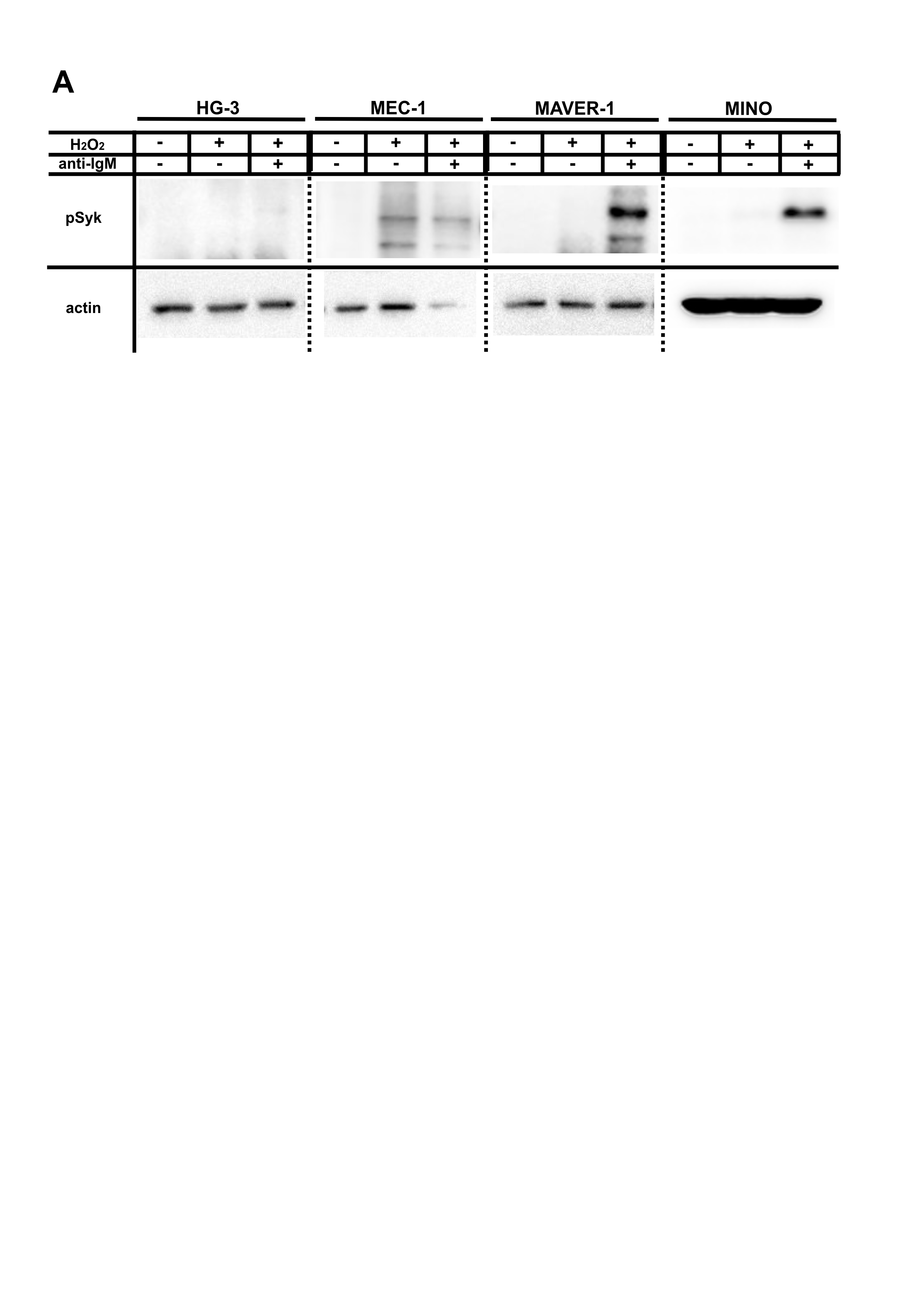

Supplement: Supplementary file 13 [file Image1.JPEG]

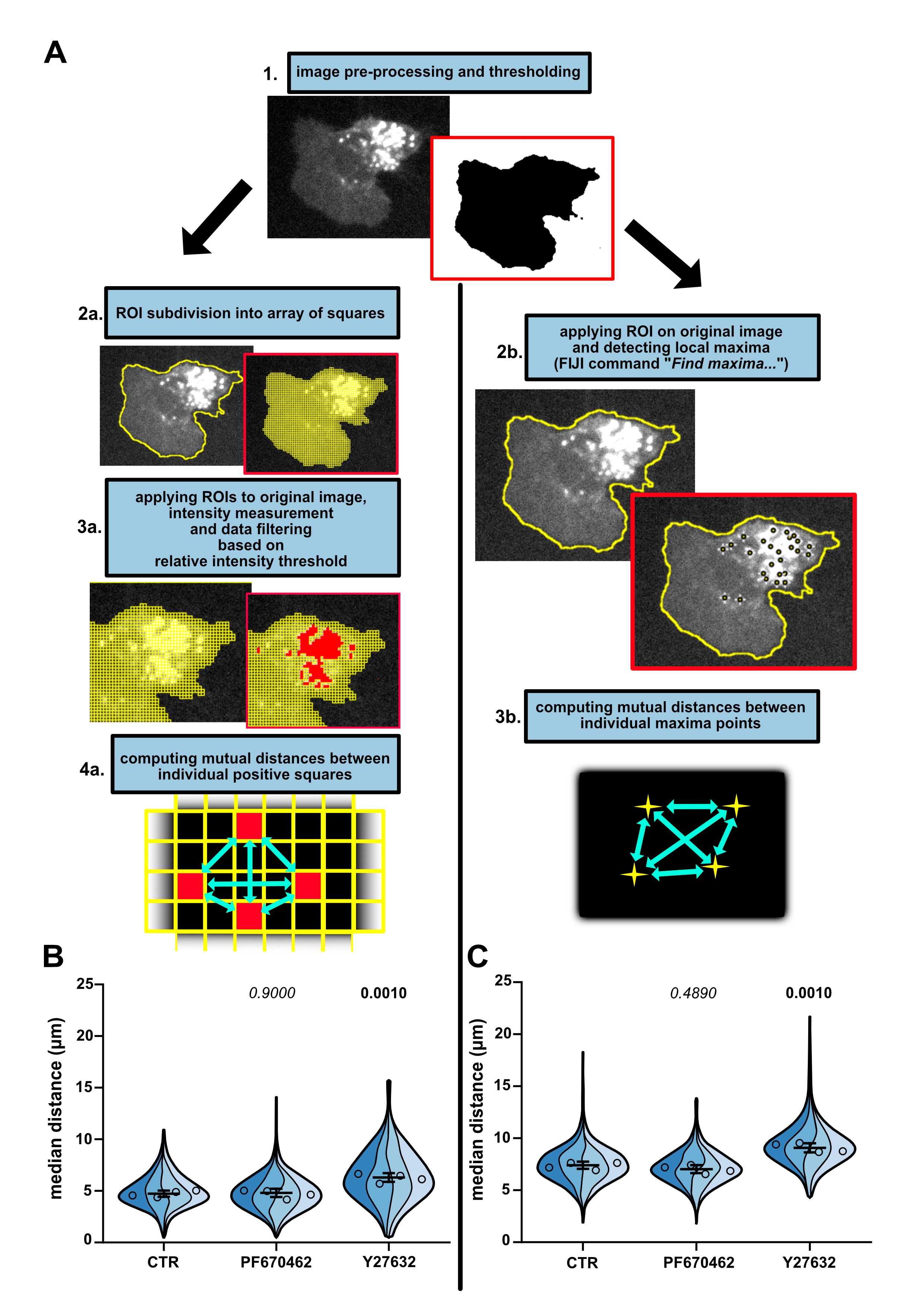

Supplement: Supplementary file 15 [file Image4.JPEG]

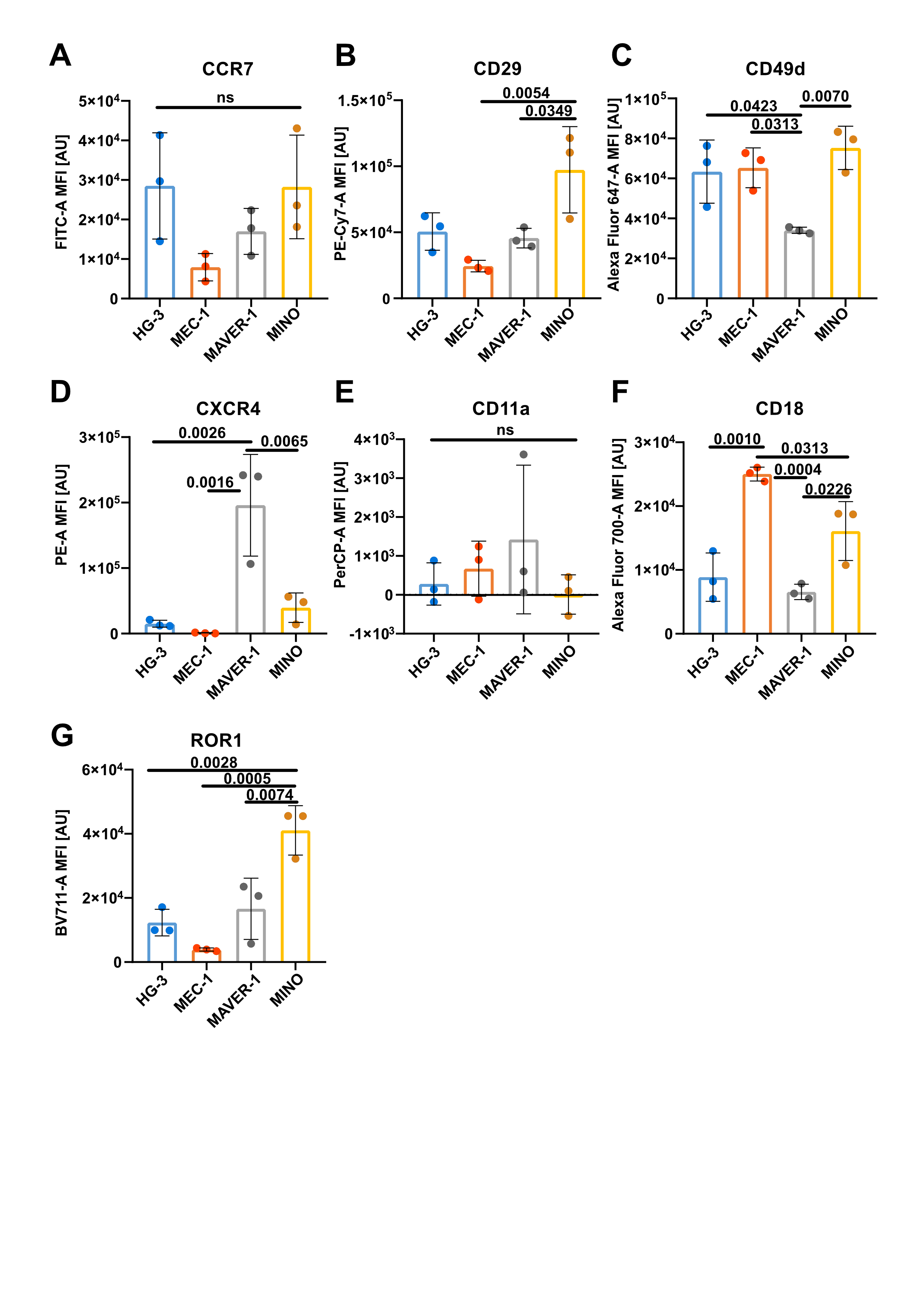

Supplement: Supplementary file 16 [file Image7.JPEG]

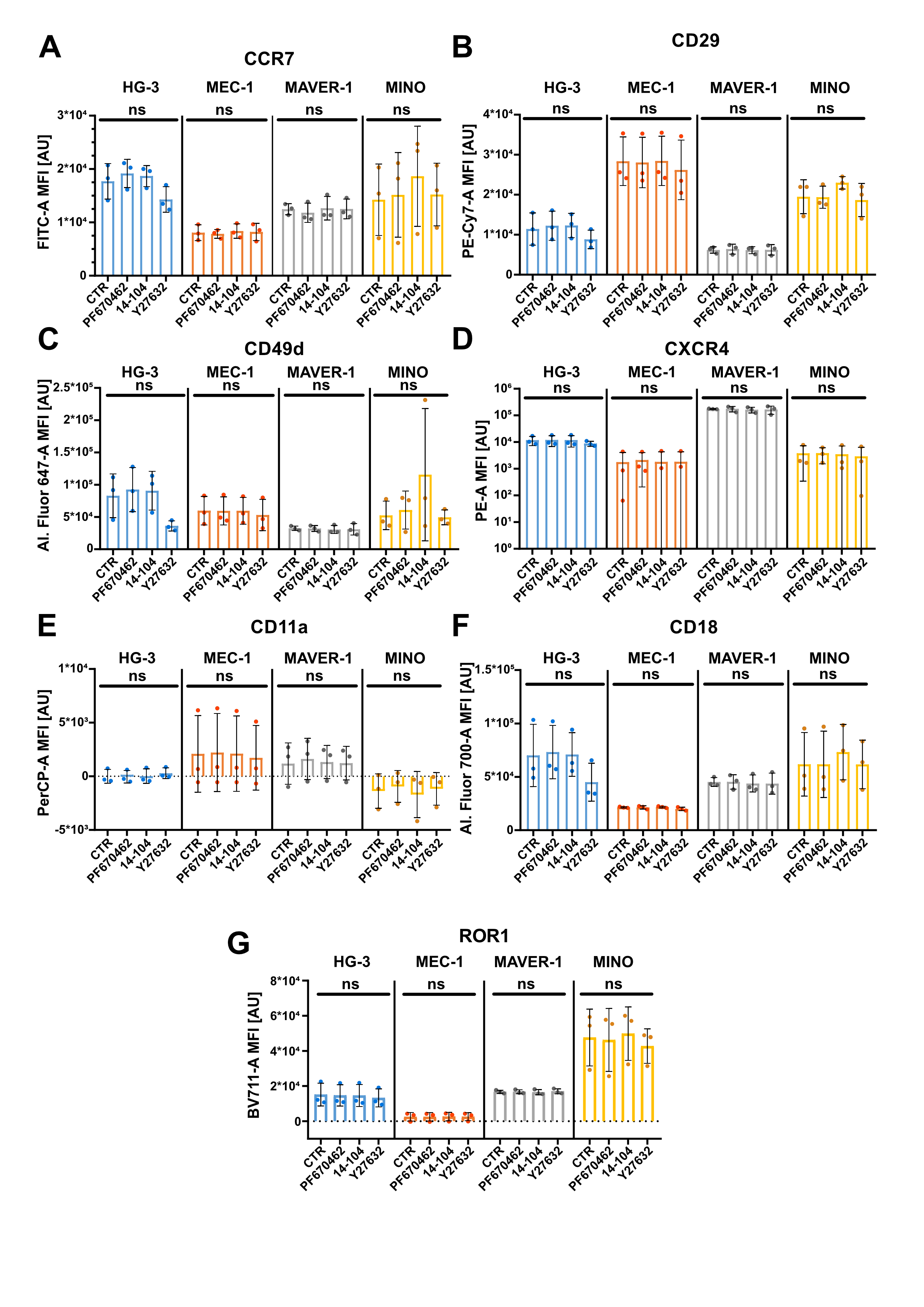

Supplement: Supplementary file 21 [file Image8.JPEG]

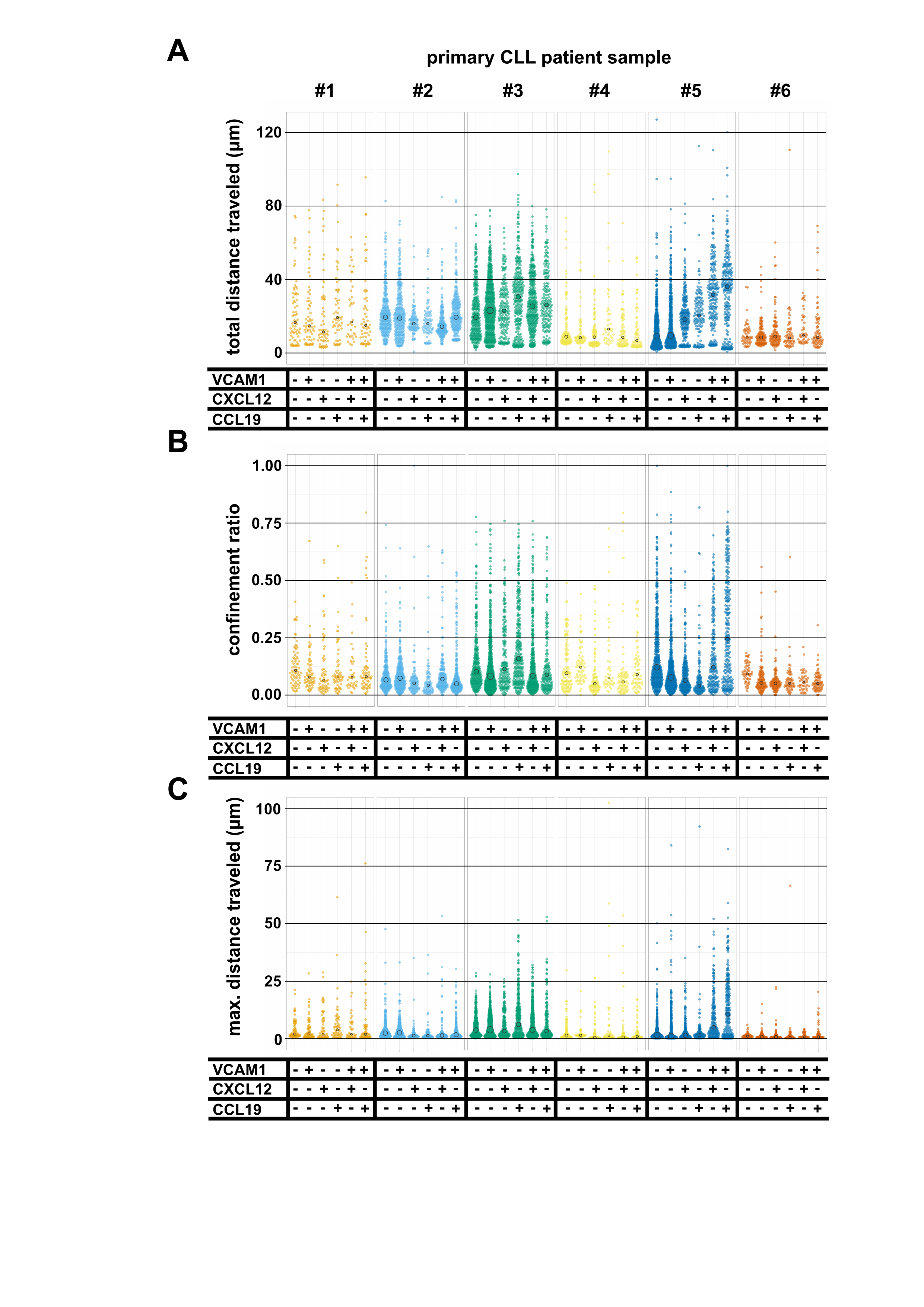

Supplement: Supplementary file 27 [file Image6.JPEG]
