# Supplementary material for: Role of casein kinase 1 in the amoeboid migration of B-cell leukemic and lymphoma cells: A quantitative live imaging in the confined environment
Source: Front Cell Dev Biol. 2022 Dec 6;10:911966. doi: 10.3389/fcell.2022.911966 (PMC9763939; doi:10.3389/fcell.2022.911966)
Supplement: Supplementary file 9 [file Table1.DOCX]

| **Reagent type** | **Designation** | **Species** | **Company** | **Identifier** | **Dilution** |
| --- | --- | --- | --- | --- | --- |
| Antibody | Anti-human CCR7 (CD197)-FITC | mouse | Sony Biotechnology | 2366075 | 1:100 |
| Antibody | Anti-human CXCR4 (CD184)-PE | mouse | Sony Biotechnology | 2132525 | 1:100 |
| Antibody | Anti-human CD11a-PerCP | mouse | Sony Biotechnology | 2353040 | 1:100 |
| Antibody | Anti-human CD18-AlexaFluor 700 | mouse | Sony Biotechnology | 2110620 | 1:100 |
| Antibody | Anti-human CD29-PE-Cy7 | mouse | Sony Biotechnology | 2115130 | 1:100 |
| Antibody | Anti-human CD49d-AF647 | mouse | Exbio | MCA2503A647 | 1:100 |
| Antibody | Anti-human ROR1-Brilliant Violet 711 | mouse | Sony Biotechnology | 2389060 | 1:50 |
