# Supplementary material for: Role of casein kinase 1 in the amoeboid migration of B-cell leukemic and lymphoma cells: A quantitative live imaging in the confined environment
Source: Front Cell Dev Biol. 2022 Dec 6;10:911966. doi: 10.3389/fcell.2022.911966 (PMC9763939; doi:10.3389/fcell.2022.911966)
Supplement: Supplementary file 17 [file Table2.DOCX]

## 1. FIJI macro for preprocessing of images for Trackmate tracking analysis (Java)

*//run("Properties...", "channels=2 slices=1 frames=60 pixel_width=0.7381885 pixel_height=0.7381885 voxel_depth=1.0000000 frame=[10 sec]");*

Stack.setChannel(2)

rename("Merged");

run("Channels Tool...");

Stack.setDisplayMode("composite");

run("Blue");

selectWindow("Merged");

run("Brightness/Contrast...");

resetMinAndMax();

*//run("Make Substack...", "channels=1-2 frames=1-60");*

run("Stack to RGB", "frames");

run("Image Stabilizer", "transformation=Translation maximum_pyramid_levels=1 template_update_coefficient=0.90 maximum_iterations=200 error_tolerance=0.0000001 output_to_a_new_stack");

run("Split Channels");

selectWindow("Merged");

*//run("Set Scale...", "distance=1.5385 known=1 unit=micron global");*

*//selectWindow("Merged");*

run("Close" );

selectWindow("Stablized Merged (red)");

run("Close");

selectWindow("Stablized Merged (green)");

run("Close");

selectWindow("Stablized Merged (blue)");

run("TrackMate");

## 2. FIJI macro for segmentation of cellular outlines from CellBrite signal (Python)

from ij import IJ

imp = IJ.getImage();

IJ.run("Duplicate...", " ");

IJ.run(imp, "Enhance Contrast...", "saturated=0.35 equalize");

IJ.run(imp, "Gaussian Blur...", "sigma=2");

IJ.setAutoThreshold(imp, "Huang dark");

IJ.run(imp, "Convert to Mask", "");

IJ.run(imp, "Dilate", "");

IJ.run(imp, "Erode", "");

IJ.run(imp, "Fill Holes", "");

IJ.run(imp, "Analyze Particles...", "size=50-Infinity clear add");

rm.select(0);
