# Supplementary material for: Role of casein kinase 1 in the amoeboid migration of B-cell leukemic and lymphoma cells: A quantitative live imaging in the confined environment
Source: Front Cell Dev Biol. 2022 Dec 6;10:911966. doi: 10.3389/fcell.2022.911966 (PMC9763939; doi:10.3389/fcell.2022.911966)
Supplement: Supplementary file 19 [file Table3.DOCX]

## 1. R-script for computation of mutual distances between positive squares (approach #1)

**Required libraries:**

{r libraries, include=FALSE}

library(dplyr)

**Loading of dataset from .csv file, data filtering based on relative intensity (more than 2x minimum median intensity):**

{r loading}

table1 <- read.csv('D:/Data/data.csv')

table2 <- table1 %>% filter(Median > (min(Median)*2) )

**Making of combinatorial matrix:**

{r comb_matrix}

rowIndices <- t(combn(nrow(table2), 2))

rowIndices <- as.matrix(rowIndices)

rowIndices <- as.data.frame(rowIndices)

V1 <- rowIndices$V1

V2 <- rowIndices$V2

**Computation of all mutual distances between any 2 squares based on coordinates of their centroids:**

{r distances}

for (i in 1:nrow(rowIndices)) {

row1 = rowIndices$V1[i]

row2 = rowIndices$V2[i]

rowIndices$X_row1[i] = table2$X[row1]

rowIndices$X_row2[i] = table2$X[row2]

rowIndices$Y_row1[i] = table2$Y[row1]

rowIndices$Y_row2[i] = table2$Y[row2]

rowIndices$distance[i] <- sqrt(((table2$X[row1]-table2$X[row2])^2)+(table2$Y[row1]-table2$Y[row2])^2)

}

**Saving the table with results:**

{r saving}

write.csv(rowIndices, "squares_data_1.csv")

**Printing the median value of calculated distances (used for statistical analysis) and the value of filtering threshold:**

{r print_median}

print(median(rowIndices$distance))

print(min(table1$Median)*2)

## 2. R-script for computation of mutual distances between local maxima (approach #2)

**Required libraries:**

{r libraries, include=FALSE}

library(dplyr)

**Loading of dataset from .csv file:**

{r loading}

table1 <- read.csv('D:/Data/data.csv')

**Making of combinatorial matrix:**

{r comb_matrix}

rowIndices <- t(combn(nrow(table1), 2))

rowIndices <- as.matrix(rowIndices)

rowIndices <- as.data.frame(rowIndices)

V1 <- rowIndices$V1

V2 <- rowIndices$V2

**Computation of all mutual distances between any 2 maxima based on their coordinates:**

{r distances}

for (i in 1:nrow(rowIndices)) {

row1 = rowIndices$V1[i]

row2 = rowIndices$V2[i]

rowIndices$X_row1[i] = table1$X[row1]

rowIndices$X_row2[i] = table1$X[row2]

rowIndices$Y_row1[i] = table1$Y[row1]

rowIndices$Y_row2[i] = table1$Y[row2]

rowIndices$distance[i] <- sqrt(((table1$X[row1]-table1$X[row2])^2)+(table1$Y[row1]-table1$Y[row2])^2)

}

**Saving the table with results:**

{r saving}

write.csv(rowIndices, "maxima_data_1.csv")

**Printing the median distance value (used for statistical analysis:**

print(median(rowIndices$distance))
